# Design and Evaluation of Ecological Interface of Driving Warning System Based on AR-HUD

**DOI:** 10.3390/s24248010

**Published:** 2024-12-15

**Authors:** Jun Ma, Yuhui Li, Yuanyang Zuo

**Affiliations:** 1 College of Design and Innovation, Tongji University, Shanghai 200092, China; majun.tongji@foxmail.com (J.M.); yottoli@foxmail.com (Y.L.); 2 School of Automotive Studies, Tongji University, Shanghai 200092, China

**Keywords:** AR-HUD, driving warning system, cognitive load, ecological interface design, autonomous driving

## Abstract

As the global traffic environment becomes increasingly complex, driving safety issues have become more prominent, making manual-response driving warning systems (DWSs) essential. Augmented reality head-up display (AR-HUD) technology can project information directly, enhancing driver attention; however, improper design may increase cognitive load and affect safety. Thus, the design of AR-HUD driving warning interfaces must focus on improving attention and reducing cognitive load. Currently, systematic research on AR-HUD DWS interfaces is relatively scarce. This paper proposes an ecological interface cognitive balance design strategy for AR-HUD DWS based on cognitive load theory and environmental interface design theory. The research includes developing design models, an integrative framework, and experimental validation suitable for warning scenarios. Research results indicate that the proposed design effectively reduces cognitive load and significantly decreases driver response and comprehension times, outperforming existing interfaces. This design strategy and framework possess promotional value, providing theoretical references and methodological guidance for AR-HUD warning interface research.

## 1. Introduction

With the increasing number of cars in the world and the increasingly complex traffic environment, driving safety has become an urgent issue. Because the current autonomous driving technology is still not very mature, nor has it obtained drivers’ full trust, the traffic warning system that senses and transmits environmental information through the car sensor combined with the driver’s response operation will become a long-term mainstream intelligent assisted driving scenario. When the driver receives information, the driving workload will be generated, including visual, auditory, mental, and cognitive aspects. It has been shown that drivers can obtain 80% of information through vision when looking at the road [1]. Therefore, providing enhanced driving safety information that matches the actual environment when the driver is looking ahead will help the driver identify the information more naturally and focus on safe driving. Head-up displays (HUDs) are currently widely used in cars. They can significantly improve the driver’s reaction time to dangerous situations in low visibility [2], which is conducive to focusing the driver’s cognitive resources on important information and reducing redundant behaviors such as lowering the head, with short information acquisition time [3,4]. On this basis, the combination of HUDs and augmented reality technology, namely AR-HUDs [5], can place visual information and prompts directly in the driver’s line of sight [6], speed up the visual processing of instant information [7], and thus reduce the potential risk caused by distraction [8]. In addition, the combination of the AR-HUD system and traffic warning can improve the efficiency and reaction speed of drivers in obtaining warning information [9] to avoid traffic accidents caused by insufficient response.

However, improper interface design of AR-HUDs can also have negative effects on cognitive resources. With the improvement of on-board computing power and network technology, the types of information displayed on an AR-HUD are increasing and becoming richer. It not only provides necessary driving information but also vehicle navigation, communication information, and multi-media content [10]. Since the display area of the AR-HUD overlaps significantly with the driver’s road field of vision, if the virtual image information projected in the driver’s field of vision is chaotic and the projected area is large, it may interfere with the driver’s perception of the real driving environment, resulting in more serious cognitive load and distraction [11]. However, an overly simple interface will also lead to unclear information transmission, and the driver will think that the AR-HUD is not helpful [12]. Therefore, it is necessary to effectively filter the information identified by the system, balance the cognitive load of the AR-HUD interface, and improve the speed and accuracy of information acquisition by users.

### 1.1. Related Research

#### 1.1.1. AR-HUD Information Interface Design

An AR-HUD combines virtual reality with the actual scenario to provide near-field and far-field warning information, vehicle information, surrounding object information, pedestrian information, navigation information, and automatic driving information to provide drivers with an immersive and intelligent driving experience. The interface of the AR-HUD is usually designed according to the principles of human–computer interaction in the car. In studies on the correlation between information presentation on AR-HUDs and cognitive load, Hooey [13] and Liu et al. [7] proved that HUDs had no obvious advantage in displaying navigation information, and HUDs can significantly accelerate the driver’s reaction time, which was more effective than a dashboard display. Okabayashi et al. [14] and Wickens et al. [15] proposed that HUDs had more obvious advantages in displaying information that required rapid response, while for information that was not urgent, the use of HUDs may more easily distract the driver’s attention. In terms of the specific form of AR-HUDs, Kim et al. [16] proved through experiments that the spatial information provided by conformal graphics on AR-HUDs can not only lead to better driving performance but also make braking behavior more stable. In another study by Kim and Gabbard [17], the AR-HUD interface could enhance the driver’s situational awareness, but it may also distract the driver’s attention. In the study of the design space of AR-HUDs, Tonnis et al. [18] defined six categories of AR information presentation principles. Kunze et al. [19] used AR to display the uncertainty of the driver assistance system, comparing 11 visual variables and finding that tone was particularly well suited to communicating emergency commands. Hauslschmidd et al. [20] defined a design space for AR applications on windshields and proposed a comprehensive and general description of the possibilities available when designing user interfaces. Wiegand et al. [21] extended this design space by showing more dimensions of the in-car AR application design space, with more options for each dimension. These approaches focus more on the behavior and relationship to the physical world but are still missing a description of the function and intent of the element. Müller et al. [22] proposed a visual classification of AR information elements covering automatic driving and manual driving and classified AR elements from four dimensions: information type, function type, content reference type, and registration type. According to the above studies, AR-HUD technology can help reduce cognitive load by providing only necessary information to drivers, so that they can focus on the main task of driving. Therefore, displaying warning information related to driving safety on HUDs is an optimal choice [23]. Although the specific warning display form on AR-HUDs has been preliminarily explored, no systematic design system and scheme have been proposed in theory and practice. In the aspect of AR-HUD design space, some systematic discussions have been put forward, but there is a lack of specific research on the design strategy of the warning system. At present, most research on automotive design is based on traditional human–machine interaction principles, and the application of other design theories in AR-HUD design, such as cognitive load theory [24] and ecological interface design theory [25], is rarely discussed. Although cognitive load is often used as an experimental assessment method (such as the NASA-TLX scale [26]), its application to automotive interfaces, especially AR-HUD interfaces, is still in its infancy and has great research potential. In addition, Kim et al. [27] applied ecological interface design to an AR-HUD for the first time, demonstrating its potential in reducing driver cognitive load and reducing distraction.

#### 1.1.2. Driving Warning System

In the research on the interface of traffic warning information related to AR-HUDs, the presentation scheme and design principle of information are explored from different views. Yang [28] analyzed and summarized the cognitive characteristics of the AR-HUD interface, analyzed the existing information importance, color formality, interface element quantity, and other issues of AR-HUDs from the perspective of road safety prompt information presentation, and put forward the principles of interface design of AR-HUD road safety prompt information. Kim et al. [5] proposed an AR-HUD interface for a collision warning system and verified that, compared with traditional DVIs, the HUD interface can make drivers realize the danger faster and significantly shorten the reaction time to the danger ahead. Based on AR-HUDs, Tran et al. [29] designed a left-turn auxiliary decision system at an intersection that displayed the predicted trajectory of the oncoming vehicle within the driver’s field of vision and helped the driver make left-turn decisions. Through experiments, they found that the use of projected trajectory AR-HUDs can enhance driver perception and reduce visual distraction. Tonnis et al. [30] studied different braking trajectory display schemes of AR-HUDs and found that the visual assistance system with a horizontal bar superimposed on the braking endpoint of the road surface to indicate the braking distance performed better overall and reduced the cognitive burden of users. He et al. [31] suggested that providing surrounding traffic information to drivers of autonomous vehicles, in addition to TOR and automation capability information, could help drivers anticipate potential traffic conflicts (early warnings), although not in vehicles with AR-HUDs. At present, in the field of the combination of driving warning systems and AR-HUDs, most of them are visual designs for single-warning information, and the presentation of multi-warning information in complex scenarios is not proposed.

#### 1.1.3. Ecological Interface Design

The ecological interface design (EID) proposed by Rasmussen and Vicente [25] is based on abstract hierarchical analysis (AH) and skill, rule, knowledge (SRK) classification. Through AH, it is possible to determine which kinds of information should appear on the ecological interface of the system [25]. Combining SRK classification to analyze the level and structure of the driver’s task, it can determine how the information should be presented [32]. Lee et al. [33] proved through experiments that, compared with the traditional interface, the driver assistance system with ecological interface design can improve the driver’s judgment accuracy and confidence, and when the participant can only view the situation in a short time, the ecological interface can make the driver understand more quickly than the traditional interface. Kim et al. [27] applied ecological interface design to AR-HUDs for the first time to design the interface of a pedestrian collision alarm system and proved through rapid prototyping that the warning display mode of projecting virtual shadow based on ecological interface design was better than the traditional boundary box marking of pedestrian warning in terms of visibility, attention, situational awareness, and so on. Schewe et al. [34] designed an ecological interface for speed control using the ecological interface design method and proved through experiments that, compared with traditional speedometers, eco-interface-designed speedometers can reduce cognitive load and help drivers better control speed and maintain lane. Schewe et al. [35] compared 2D and 3D visual effects speedometers based on HUDs and applied ecological interface design. Through experimental evaluation, they found that a 2D ecological interface speedometer could help users control speed more accurately, while a 3D ecological interface speedometer could help users keep lanes more accurately. Cao and Zhang [36] used the SRK classification method to analyze the task hierarchy and structure of urban rail drivers and further introduced the display information content and structure of an urban rail AR-HUD based on it. In general, the current relevant studies have proved that the application of EID in the AR-HUD interface has great research prospects and potential to help drivers reduce cognitive load and reduce attention distraction. However, at present, the number of studies on the ecological interface design of AR-HUDs is still limited, and most of them only start from a single scenario to verify the effectiveness of a certain ecological interface design scheme and have not yet proposed a systematic ecological interface design strategy for the current normal and complex traffic scenarios.

#### 1.1.4. Measurement and Evaluation Methods of Driving Cognitive Load

Driving cognitive load measurement methods include subjective methods and objective methods (i.e., physiological measurement methods), and driving performance can be used as a method to assess the impact of cognitive load [37]. Physiological indicators can also be used as monitoring indicators of driving cognitive load because these indicators can be collected in real time during the completion of driving tasks. The subjective workload scales commonly used to measure driver workload (DWL) include the NASA Task Load Index (NASA-TLX) scale, the Driving Activity Load Index (DALI) scale, the Subjective Workload Assessment Technique (SWAT) scale, and the Mental Labor Rating Scale (RSME). DALI is a workload indicator proposed by Pauzie [38] based on driving characteristics and further developed on the basis of NASA-TLX, including five dimensions of attention: visual demand, auditory demand, time demand, interference, and situational pressure. Objective measurement methods usually refer to physiological measurement methods. The indicators used in physiological measurements are mainly divided into three categories: cardiac activity indicators [39,40], eye-tracking analysis indicators [41,42], and brain imaging analysis indicators [43,44]. The eye-tracking analysis index has advantages in simulated driving experiments and is widely used in the research field of autonomous driving and driving analysis [45,46]. Lu and Sarter [47] suggested that eye tracking is an effective technique for inferring changes in operator trust levels in real time. Compared to other physiological measures, eye tracking has the benefits of easier implementation, less intrusion, and a more fine-grained analysis of monitoring behavior. Drivers’ excessive trust in the driving automation system will lead to less attention on the road [48,49,50].

### 1.2. Research Questions and Article Structure

Based on the cognitive load theory, this study summarized the cognitive process and characteristics of driving warning systems (DWSs), combined with the ecological interface design method, and proposed a systematic design strategy to optimize the interface presentation mode of the AR-HUD DWS to balance the interface cognitive load, improve the information receiving efficiency, and improve driving safety. To achieve our research objectives, we need to answer the following research questions:

RQ: How to combine the ecological interface design method to build the interface design strategy of an AR-HUD DWS to balance the interface cognitive load and improve driving safety?
SQ 1: How to balance the cognitive load of an AR-HUD DWS?
SQ 2: How to build a cognitively balanced AR-HUD DWS ecological interface design interface?
SQ 3: Can the constructed AR-HUD DWS interface cope with complex-warning scenarios to improve driving safety?

Based on the above issues, the work in this article proceeds on the following levels:

To address SQ 1, we applied the AH and SRK classification in the ecological interface design theory to the analysis of the AR-HUD DWS and proposed a cognitive adjustment method of the warning interface based on the cognitive load theory. Combined with the characteristics of the AR-HUD warning interface, the design dimension was analyzed. Finally, the strategy and model of the EID of the AR-HUD driving warning system were constructed from the system layer, strategy layer, and vision layer.

To address SQ 2, an integrated AR-HUD interface design framework was designed based on the EID strategy of the AR-HUD DWS. This framework was suitable for different warning scenarios, including horizontal and vertical control, with or without obstacles, single-warning scenarios and complex-warning scenarios. The AR-HUD warning interface prototype was designed for five different warning scenarios.

To address SQ 3, based on the experimental design of five different warning scenarios, combined with subjective and objective measurement methods of cognitive load and risk reaction time, we evaluated the cognitive workload and warning reaction time of drivers in single-warning and complex-warning scenarios. Finally, the validity of the AR-HUD interface design strategy and framework was analyzed and verified by measuring data, and the key application advantages and design strategy methods were summarized.

The structure of this paper is as follows: in Section 2, the cognitive balance design strategy and model of the ecological interface of the AR-HUD DWS is constructed, and, further, the ecological interface design framework of the AR-HUD DWS is built. In Section 3, a static driving simulation platform is built, and the AR-HUD interface design strategy and the effectiveness of the framework are tested through different warning scenario experiments. In Section 4, we discuss the experimental results and discuss the limitations of this study. Finally, the research work of this paper is summarized.

## 2. Theoretical Framework

Based on the above problems, this paper analyzes and summarizes the relevant theoretical basis from four aspects, as shown in Table 1. The theoretical analysis consists of four main parts: (A) principle and constitution of DWS, (B) features of AR-HUD-based driving warning system, (C) cognitive load theory (CLT), and (D) ecological interface design (EID).

The details are explained as follows: first, we analyzed the principle and constitution of DWS, including its basic working principle (A-1), constituent components (A-2), and graded warning mechanisms (A-3), which clarified the structure of the warning system. Next, we conducted an in-depth analysis of the AR-HUD principle (B-1), examined different warning interface cases (B-2), and summarized the characteristics of AR-HUD warning interface design (B-3). Then, we analyzed cognitive load theory, covering its basic concepts (C-1), the different types of cognitive load (C-2), and the application of cognitive load in the driving domain (C-3). Finally, we explored the theory and methods of ecological interface design (D-1), including abstract level analysis (D-2) and the SRK classification framework (D-3), as well as how to effectively transform interface design elements through ecological interface semantic mapping. These theories provide crucial design guidance for creating an intuitive and efficient AR-HUD driving warning interface. The theoretical analysis provides a solid theoretical foundation for the interface design and establishes evaluation standards for the construction of a cognitively balanced AR-HUD driving warning system.

### 2.1. Ecological Interface Cognitive Balanced Design Strategy

**Figure 1:**
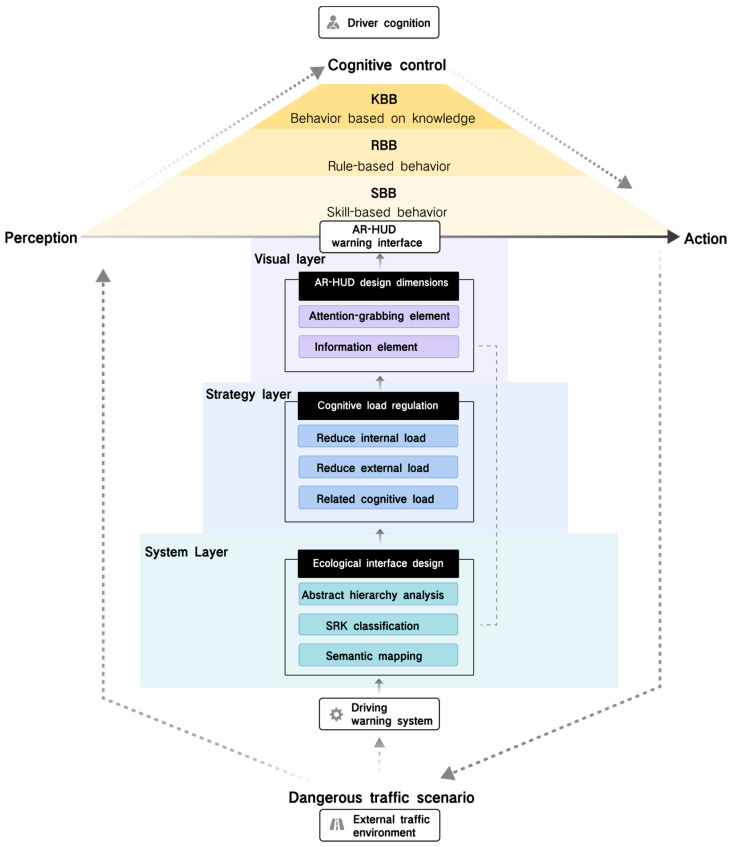
The design strategy of ecological interface cognitive balance of AR-HUD DWS.

According to the research of DWS, AR-HUD interface design, CLT, and EID method, three layers of a balanced cognitive load AR-HUD warning interface can be constructed, as shown in Figure 1. Firstly, at the system layer, the external environment and the architecture of the warning system are analyzed through ecological interface design. Secondly, at the strategy layer, the interface design strategy is adjusted by the causal dimension of cognitive load. Finally, at the visual layer, the design process is considered from the visual design dimension of the AR-HUD. This comprehensive dynamic analysis ensures that the warning interface meets system architecture, driver cognitive, and AR-HUD characteristic needs. The three layers are organically combined. For example, the physical form layer at the abstract level intuitively presents the function through the AR-HUD design dimension, and the SRK classification also uses the classification of these dimensions to classify the types of warning information. In addition, in terms of semantic mapping, the visual design is optimized by combining the visual thesaurus and AR-HUD warning interface. The application of these three hierarchical methods helps to balance the cognitive load, match the driver’s internal cognition with the external environment, promote the realization of skill-based behavior, make the driver react quickly in dangerous scenarios, and improve the acquisition ability of the driving schema.

*System layer: analysis of environment and system by ecological interface design.*

In the cognitive load balance design of the AR-HUD warning system, it is important to analyze and construct the relationship between the external environment and the warning system by adopting the principle of ecological interface design (EID). The goal of EID is to create an intuitive representation that makes system status and operation clear and understandable to the driver, enabling efficient decision making. First of all, through the abstract hierarchy analysis of EID, the overall framework of the AR-HUD system can be deeply understood, and the interaction and dependence of various layers in the purpose–means structure can be revealed. Secondly, the whole–part structure helps to identify the constraints on variables at each layer and explore their interactions. These variables can be mapped to the SRK classification to identify the cognitive needs of different driving tasks. Finally, semantic mapping translates these constraints and information requirements into specific interface elements to ensure that information presentation matches driver cognitive patterns. Through this series of analyses, the AR-HUD warning system interface is carefully constructed to support fast, intuitive cognitive processing to help drivers maintain cognitive load balance and optimize the overall driving experience.

*Strategy layer: interface elements of cognitive load regulation.*

In the strategy layer of the AR-HUD warning system, the goal of cognitive load regulation is to effectively transmit warning information without exceeding the processing ability of the driver. This requires careful design of interface elements to enable rapid absorption and understanding of information while reducing unnecessary cognitive load. It is necessary to comprehensively consider the nature of the task, the driver’s ability, and their interaction to reduce the internal and external cognitive load and improve the related cognitive load. To this end, a modular holistic task strategy is adopted to present information in stages to help drivers understand and process the warning information. At the same time, it is necessary to avoid unnecessary comparisons between goals and problem states, reduce information redundancy, and integrate information sources in emergencies to prevent distraction. The interface design should also be adjusted according to the professional knowledge level of the driver to provide appropriate information and support. To improve the related cognitive load, task variation design and embedded stent design can be adopted. Task variation design helps drivers identify key features and promotes schema transfer. Implicit and explicit supports, such as cues and feedback, help drivers effectively identify and respond to potential hazards. These design strategies aim to improve the driver’s cognitive processing efficiency and reduce the safety risks caused by improper information processing. In summary, the balance needs to be found between interface elements, information presentation, and user interaction, ensuring that every design decision is aimed at improving task efficiency and safety while reducing cognitive stress so that the AR-HUD warning interface is aligned with the driver’s cognitive patterns and needs.

*Visual layer: visual interface presented from the AR-HUD design dimension.*

In the visual hierarchy design of AR-HUD, the warning system should utilize augmented reality technology to effectively present visual information and match the driver’s visual perception and cognitive process. It is not only necessary to focus on the accurate transmission of information but also to consider the impact of visual presentation on the driver’s understanding and reaction. The design dimension of the AR-HUD interface should be combined with the characteristics of warning information and the display characteristics of AR technology to distinguish the attention-grabbing elements and information content elements. The visual design task is to ensure that the warning elements attract the driver’s attention while keeping the message clear and easy to read. Attention-grabbing elements include registration type, visual field position, and visual design factors. The registration types include 3D registration, 2D registration, angle simulation, and unregistered forms, which have different limitations in visual space and depth. The space and depth perception advantages of the AR-HUD should be utilized in the design to integrate warning information into the driver’s field of view to create an intuitive information layer. Visual field position determines where information is presented, while visual design factors focus on color, transparency, dynamic frequency, and saturation. Information content elements are divided into location-specific elements, dynamic environment elements, and action suggestions, corresponding to KBB, RBB, and SBB in SRK classification, respectively. Combined with the visual thesaurus of ecological interface design, we can develop visual languages that conform to cognitive habits and improve information acceptance and warning effects. In short, the interface design of the AR-HUD warning system needs to comprehensively consider the synergy of visual elements to maximize the effect of information transmission and ensure its practicality and effectiveness in warning scenarios.

### 2.2. Ecological Interface Design of AR-HUD Warning System

Based on the proposed design strategy, the information analysis, design, and construction of the AR-HUD interface of the warning system are carried out from the system layer, strategy layer, and vision layer. At the system layer, the interface information architecture is obtained from the means–purpose structure of the abstract-level analysis of the AR-HUD warning system. Combined with the whole–part structure, the key variables corresponding to the information can be associated, the SRK classification of this information can be conducted, and the types of corresponding information elements can be judged. At the strategy level, the internal cognitive load of warning information is reduced by using the modular overall task strategy. By considering different cognitive load effects and professional knowledge reversal, the external load of interface design is reduced. By changing the structure of the task itself and embedding cognitive stents to improve the related cognitive load, cognitive load regulation analysis, interface information architecture, and presentation strategy are carried out. Finally, through the integration of semantic mapping of the visual layer and AR-HUD interface design dimension analysis, the AR-HUD warning interface design framework is constructed.

According to the previous analysis of interface design dimensions, warning visual design elements are constructed from two categories: attention-attracting elements and information content elements:

1. Attention-grabbing elements.
  (1) Registration type: Three-dimensional registration is adopted to present the AR interface. Considering that the design is best presented as a whole and reflects changes in related variables, a 3D registration form that presents (x, y, z) three-dimensional coordinates in space can more intuitively show spatial variables and facilitate the integration of elements in different dimensions of space. In addition, due to the need to directly reflect the impact of driver operations on variables, the method of 3D registration is also more consistent with the driver’s cognition of changes in the real world.
  (2) Visual field position: The driver’s visual field is fixed in the foveal visual area (2° away from the normal line of sight) to present the warning interface. Since the warning scenario requires the driver’s fastest visual search speed to prevent the occurrence of dangerous accidents, fixing the warning interface in the foveal visual area can improve the driver’s attention and perception, without searching for warning tips in the field of vision.
  (3) Visual design factors: (a) Color: For the warning color, the danger-level color that is widely recognized by the public is adopted, such as orange or yellow, which represents the lesser danger degree, while red represents the most urgent danger degree, and green represents safety. At the same time, the three colors of red, yellow, and green are also consistent with the color of the traffic light, reflecting the semantic information of stop (danger), wait (attention), and pass (safety). (b) Transparency: The contrast presentation of transparency, that is, the gradient state, can map predictive information such as orientation or path. (c) Dynamic frequency: The dynamic frequency changes in real time with the change in dynamic environment monitoring to ensure that the driver’s operation can obtain immediate feedback on the interface. (d) Saturation: The higher the saturation, the darker the color, and the more critical the situation.
2. Information content element.

**Figure 2:**
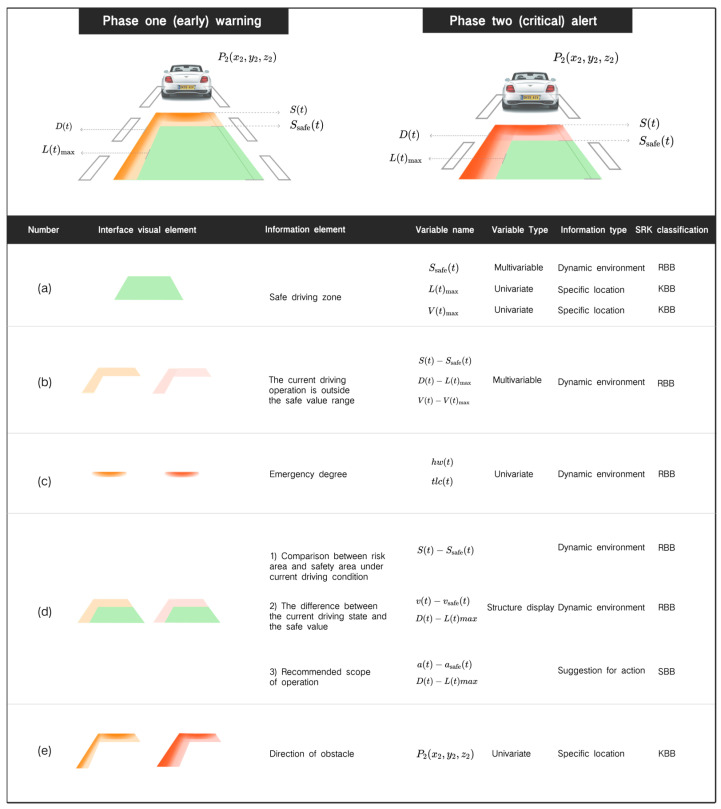
AR-HUD interface design framework of the warning system. Green represents safety and red represents danger.

At present, most AR-HUD interfaces only use one type to provide warning information, which easily leads to the imbalance of cognitive load in the warning interface. Therefore, this study not only considers the integration of the three information elements into the overall structure but also considers the presentation of different information elements to drivers with different cognitive levels, that is, the independence of the three elements. After comprehensively considering the information design architecture and principles of the above system layer, strategy layer, and vision layer, the overall AR-HUD warning interface design framework is analyzed and constructed, as shown in Figure 2 (see Supplementary Note S1 for details).

From the overall visual point of view, the AR-HUD warning interface is displayed in the shape of a “carpet”, a visual combination of multiple warning information components. The warning color of the overall classification of the interface adopts the three colors of red, yellow, and green, which are widely recognized by the public and represent the warning signals and are also three colors in the traffic lights, referring to different warning levels: green is safe, yellow is the early warning (notice) of phase one, and red is the critical warning of phase two. The interface elements are presented in combination with univariate mapping, multi-variable mapping, and structure display, which are mainly divided into three subcomponents (a–c) and two combined components (d, e). In different warning subsystems, each component represents different meanings of warning.

Figure 2a shows the safe driving area in the current traffic environment, and its width is equal to the width of the vehicle body. In different warning scenarios, the meaning of this component refers to different safety variables, and the SRK and information types are also different. For example, in collision warning, its vertical boundary value refers to $S_{safe}\left(t\right)$, the safety distance that changes over time, which is related to the difference in speed between the vehicle and obstacles, and the headway threshold HW∗. Therefore, it belongs to the RBB information type in a dynamic environment. In lane departure warning, the lateral boundary value represents the maximum allowable lateral departure ${L\left(t\right)}_{max}$, which can be directly presented on the coordinates of the driver’s perspective in combination with the 3D registration display mode of the AR-HUD and can inform the driver of the location information of the specific safety area, so it belongs to the KBB classification type.

Figure 2b indicates the range beyond the safe value under the current driving operation. The larger the transverse and longitudinal area, the more the current driving behavior (transverse and longitudinal acceleration) exceeds the safe value. The area will change in real time with the driver’s operation change and provide the change mapping of the current driving key value. For example, in the longitudinal direction, when the speed of the car is faster, the longitudinal area is larger. In the AR-HUD interface, the current displacement of the Y-axis can be estimated:

(1) $$\vec{v}\left(t\right)=\vec{v_{0}}+\int_{0}^{t}\vec{a}\left(\tau \right)d\tau ,$$

(2) $$\vec{s}\left(t\right)=\vec{s_{0}}+\int_{0}^{t}\vec{v}\left(\tau \right)d\tau ,$$

Here, $\vec{v}\left(t\right)$ is the velocity vector at time t, $\vec{v_{0}}$ is the initial velocity vector, and $\vec{a}\left(\tau \right)$ is the acceleration vector at time τ; $\vec{s}\left(t\right)$ is the displacement vector at time t, $\vec{s_{0}}$ is the initial displacement vector, and $\vec{v}\left(\tau \right)$ is the velocity vector at time τ. Therefore, the driver can perceive the change in speed according to the change of displacement, and can perceive the range of change, to properly accelerate and decelerate. It is also conducive to the driver to build a schema of real-time changes in risk factors with driving operations.

Figure 2c shows a gradual color block whose saturation increases as the current warning risk value increases, indicating the urgency of the current situation. In phase one of the warning, a light-yellow gradient appears, and as the risk level continues to increase, it gradually becomes dark yellow. If the risk increases to phase two of the warning, the dark yellow gradient changes to light red, and then continues to deepen to dark red, indicating that the warning level continues to increase.

**Figure 3:**
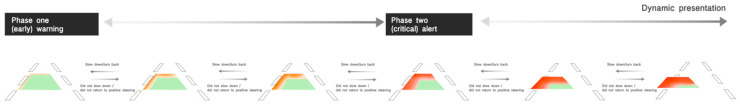
Dynamic change of warning risk. Green represents safety and red represents danger.

Combined with Figure 2b,c, the interface of dynamic change of warning risk is shown in Figure 3. When the warning risk is higher, the area in Figure 2b is larger (that is, the area in Figure 2e), and the saturation in Figure 2c is higher.

Because the driver’s horizontal and vertical operations can change the current risk state in real time, the critical risk may also revert to the early risk, and the area will decrease and the saturation will decrease until it gradually disappears, showing a dynamic change process. At the same time, the visual changes in area and color saturation in Figure 2b,c also promote the driver to understand the change in risk under dynamic operation and help the driver to construct the relevant cognitive load of dangerous scenarios.

Figure 2d is a composite component of Figure 2a,b, and there are three main information elements that can be reflected: the comparison between the risk area and the safety area under the current driving state; the difference between the current driving condition and the safe value; recommended scope of operation. In the current driving state, the risk area and the safety area shown in Figure 2d are the combined components of (a) and (b), and the comparison between the two can reflect the gap beyond the safety range under the current driving operation. Since (b) will change in real time with the driver’s operation, if (b) continues to shrink in the direction of (a) and the area continues to decrease (as shown in Figure 3), it means that the change in the current driving behavior reduces the risk; otherwise, it means that the risk continues to increase. In the information element of the difference between the current driving state and the safety value, as (d) is the real-time feedback and intuitive mapping of the difference between the safety value and the driving behavior, it can be used as the type of action suggestion information to stimulate the SBB and inform the driver of the direction of safe operation, as shown in Figure 2, that is, (b) in the direction of (a) contraction, and the longitudinal (Y-axis) contraction represents deceleration. Lateral (X-axis) contraction represents a return to positive steering. This visual schema allows the driver to perceive the association between the current driving operation and the change in the hazard level through appropriate operation and reduces the risk of an accident by controlling the area operation of the value (b). In the matching of the suggested operation range information elements, the visual effect can also inform the driver of the reasonable operation range, as shown in Figure 2, that is, (b) shrinks in the direction of (a) until the area is 0 and disappears, the warning is lifted, and the effect shown in (a) is achieved (all-green trapezoid). Figure 2e is a composite component of Figure 2b,c). The orientation and transparency changes in the visual element in Figure 2c represent the direction of the obstacle. The derivation process is as follows:

The direction vector of the obstacle is the vector from the observation point $P_{1}\left(x_{1},y_{1},z_{1}\right)$ to the obstacle $P_{2}\left(x_{2},y_{2},z_{2}\right)$, which can be represented by $P_{2}-P_{1}$, as follows:

(3) $$\vec{d}=\left(x_{2}-x_{1},y_{2}-y_{1},{\;z}_{2}-z_{1}\right).$$

Here, we take the unit vector to represent the direction of the obstacle:

(4) $$\vec{u}=\frac{\left(x_{2}-x_{1},y_{2}-y_{1},z_{2}-z_{1}\right)}{\sqrt{{\left(x_{2}-x_{1}\right)}^{2}+{\left(y_{2}-y_{1}\right)}^{2}+{\left(z_{2}-z_{1}\right)}^{2}}}.$$

The display area in Figure 2c does not exceed the area in Figure 2e, which represents the relationship between the collision risk of the obstacle and the current speed. When the distance of the obstacle is closer, the area in Figure 2c occupies a larger proportion in Figure 2b. If the speed changes to a safe speed, that is, Figure 2b gradually shrinks in the direction of Figure 2a until it disappears, then Figure 2e, representing the direction, will also disappear simultaneously, which is conducive to constructing a relationship schema between obstacle distance and driving operation for the driver.

## 3. Methods

### 3.1. Hypotheses

The research objective of this paper is to propose an AR-HUD warning interface design strategy and framework that can balance cognitive load based on the ecological interface design method, and based on this, produce an AR-HUD warning ecological interface that can effectively reduce the driver’s cognitive load and reaction time, which is suitable for different single-warning scenarios and complex-warning scenarios. The following hypotheses are proposed:

**H1:**  *The AR-HUD warning ecological interface can reduce the driver’s cognitive workload in different single-warning scenarios.*

**H2:**  *The AR-HUD warning ecological interface can also reduce the driver’s cognitive workload in complex multi-warning scenarios.*

**H3:**  *Whether in single-warning or multi-warning scenarios, the AR-HUD warning ecological interface can reduce the driver’s risk perception and decision time.*

### 3.2. Participants

According to the reports [51,52], automobile consumer groups showed a younger trend, and consumers under the age of 35 accounted for more than 40%; the post-1995 generation was the main consumer of intelligent connected vehicles and autonomous vehicles. Therefore, 25 young participants aged 22 to 27 were recruited through social media promotion. They were required to hold driving licenses to ensure that they had an in-depth understanding of China’s road traffic laws and regulations. All participants had at least one year of driving experience and drove an average of more than 100 km per week. The participants were required to have driving experience in autonomous vehicles (L1–L3) and own or permanently drive an autonomous vehicle equipped with a HUD display. They worked in a wide range of industries, including engineering, economics, and the humanities, excluding those in the automotive and design industries. Table 2 shows the basic information of participants. They were in good physical condition with normal or corrected vision. Each participant signed an informed consent form before participating in the experiment; the average duration of participation was approximately 30 min, and participants were paid after participation. This study was conducted following the Declaration of Helsinki and approved for human research by the Science and Technology Ethics Committee of Tongji University (No: tjdxsr053).

### 3.3. Experimental Design

Based on the overall AR-HUD warning interface design framework, specific variables and prototypes in the warning subsystem can be deduced. We have designed the warning interface prototype for four single-warning scenarios (forward collision warning, speed limit warning, lateral collision warning, lane departure warning), and one complex scenario: lane departure warning and lateral collision warning occurring simultaneously (see Supplementary Note S2 for details). These scenarios can cover the driver’s horizontal and vertical warning, dynamic and static obstacle warning, and traffic control warning scenarios.

#### 3.3.1. Warning Scenario

Five warning scenarios were designed, and two different AR-HUD warning interfaces were presented in the warning scenarios. These five scenarios contained different warning information and control situations in driving, including horizontal and vertical observation or operation, with or without obstacles, and single-warning or complex-warning scenarios. The experiments on these five warning scenarios can involve most situations in road warning with experimental representation and credibility.

**Figure 4:**
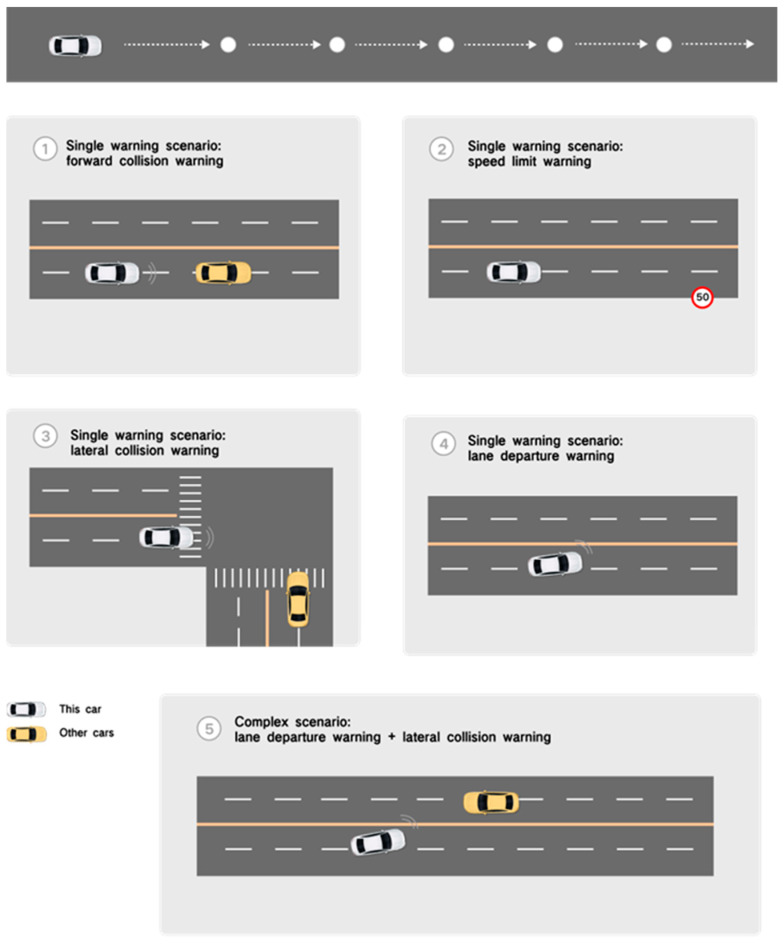
Scenario design of five warning experiments.

As shown in Figure 4, five events occurred randomly during driving to reduce driver learning effects. Each scenario took about 2 min, and the entire scenario took about 10 min. The experimental process was controlled by setting up different dangerous situations in each scenario:

(1) S1: Forward collision scenario.

The driver was instructed to drive at the specified speed of 50 km/h. If the front car decelerated on the road, there was a risk of collision between the two cars, and the driver needed to manually operate to avoid collision through warning prompts.

(2) S2: Speed limit scenario.

The driver was instructed to drive at a speed of 60 km/h and gradually approached the section of road with a driving speed limit of 50 km/h. Drivers needed to follow speed limit cues to avoid speeding.

(3) S3: Lateral collision scenario.

The driver was instructed to drive in a straight line on the road at a speed of 40–50 km/h. When entering the intersection, other vehicles were entering at the right intersection at the same time, and there was a risk of collision between the two vehicles. The driver should manually operate to avoid collision through warning prompts.

(4) S4: Lane departure scenario.

The driver was instructed to drive in the left lane at a speed of 30 km/h while maintaining a slight left-turn angle. The vehicle faced the risk of lane departure, and the driver needed to turn back in the positive direction according to the lane departure prompts.

(5) S5: Complex scenario: lane departure scenario and lateral collision scenario.

The driver was instructed to drive in the left lane at a speed of 30 km/h while keeping a slight left-turn angle, and the vehicle may face the risk of lane deviation. At the same time, when the other vehicle approached from the opposite adjacent lane, there was the risk of collision between the two vehicles. The driver should avoid lane deviation and lateral collision risk through transverse and longitudinal operation according to the warning tips.

#### 3.3.2. AR-HUD Warning Interface

**Figure 5:**
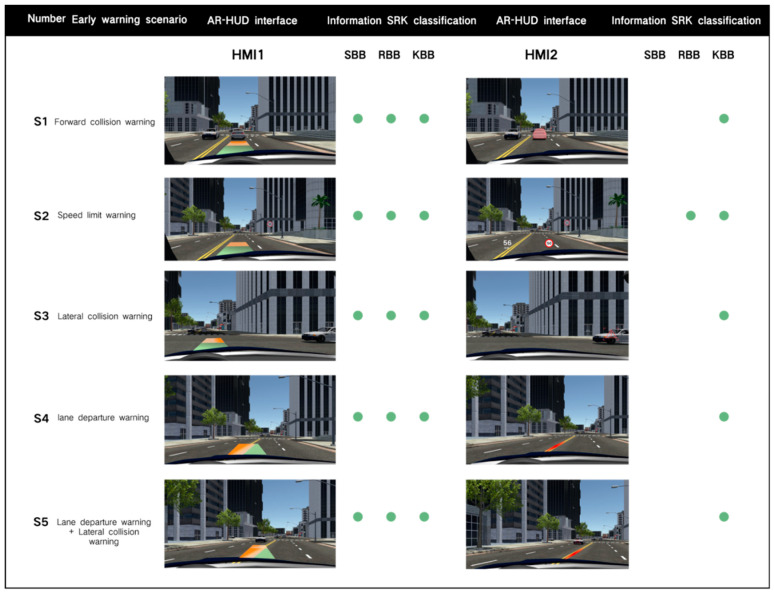
Two types of warning interfaces and information SRK classification in the five warning scenarios.

For different warning scenarios, we selected the mainstream AR-HUD warning interface (AR-HUD warning common interface) on the market as the control group. Figure 5 shows the interface and information SRK classification of the experimental group (HMI1) and the control group (HMI2) in different scenarios.

The AR-HUD warning interfaces of HMI1 contain three types of SRK classification, specific location, dynamic environment, and action suggestions. They are integrated into an integrated universal warning interface, maintaining a uniform design form in different scenarios. According to the current mainstream AR-HUD interface, the warning interface of HMI2 only contains one or two types of SRK information, most of which is KBB information. In other words, only a specific position is displayed, which theoretically requires more cognitive control and lacks the type of action advice information that can stimulate SBB behavior. To avoid grading warnings becoming an experimental variable, both HMI interfaces combine the mechanism of grading warnings, providing warning information of different danger levels, which are presented in color changes (yellow and red). Each scenario contains phase one (early) and phase two (critical) of hierarchical warning.

### 3.4. Experimental Equipment

**Figure 6:**
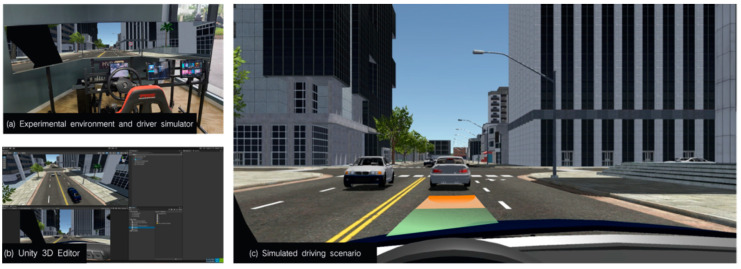
Experimental equipment and materials: (**a**) driving simulator, (**b**) Unity3D program, (**c**) simulated experimental scenario by overlaying AR visualization setup.

The experiment was conducted in the virtual driving simulation environment of the Automotive Human Factors Laboratory of Tongji University. The experimental environment consists of a fixed-base driving simulator, a main driving system, and an experimental monitoring system. The fixed-base driving simulator and the main driving system are located in the center of the experimental area, and the experimental monitoring system is located on the left side of the driving simulator, parallel to the front of the simulator, as shown in Figure 6. The experiments were performed using Unity 2023 software to create virtual driving scenarios and presented on three 55-inch monitors with a resolution of 3840 × 2160. The driver’s eye-tracking data were recorded using the Tobii Glasses 2 Head Unit, and the eye-tracking data were analyzed using the associated Tobii Pro Lab 1.194 software.

### 3.5. Dependent Variable Metric

1. Subjective Cognitive Workload: Driving Activity Load Index (DALI) Scale.

The Driving Activity Load Index (DALI) is a driving-specific modification of the NASA-TLX. Based on the same principles as NASA-TLX, DALI assesses five dimensions: attention demand, visual demand, interference level, time demand, and situational pressure. The rating scale ranges from 1 to 10, and the final score is calculated using a weighted sum of the five dimensions.

2. Objective Cognitive Workload: Eye-Tracking Data.

Eye-tracking analysis is used to investigate participants’ cognitive processes, and the data obtained are quantitatively analyzed. Since the position and appearance time of the AR-HUD in each scenario vary, the areas of interest (AOIs) are dynamically defined based on the human–machine interface (HMI) for each scenario. AOI1 is defined for the appearance time and position of HMI1, while AOI2 is defined for the appearance time and position of HMI2 in each scene. The cognitive workload is measured using two indicators: average fixation duration (AFD) and average pupil diameter (APD).

Average fixation duration (AFD): The average length of time spent fixating on a point. Longer fixation times may indicate higher cognitive processing demand. In contrast, shorter fixation times, while still allowing for effective avoidance of most accidents, suggest that the interface design does not overly distract the driver and helps in detecting potential hazards.

Average pupil diameter (APD): Larger pupil dilation typically indicates higher cognitive load, requiring the driver to allocate more attention or cognitive processing to the current information.

3. Risk Response Time

Risk response time is divided into two components: risk perception time and risk decision time [53]. Risk perception time refers to the time difference between the appearance of the risk and the participant’s awareness of it. Risk decision time is the time taken by the participant to provide feedback after perceiving the risk.

**Figure 7:**
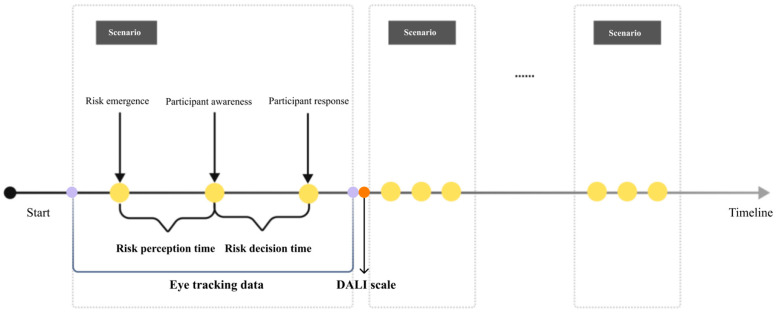
Diagram of dependent variable data extraction periods.

The detailed data extraction periods for these three indicators are shown in Table 3 and Figure 7.

### 3.6. Procedure

**Figure 8:**
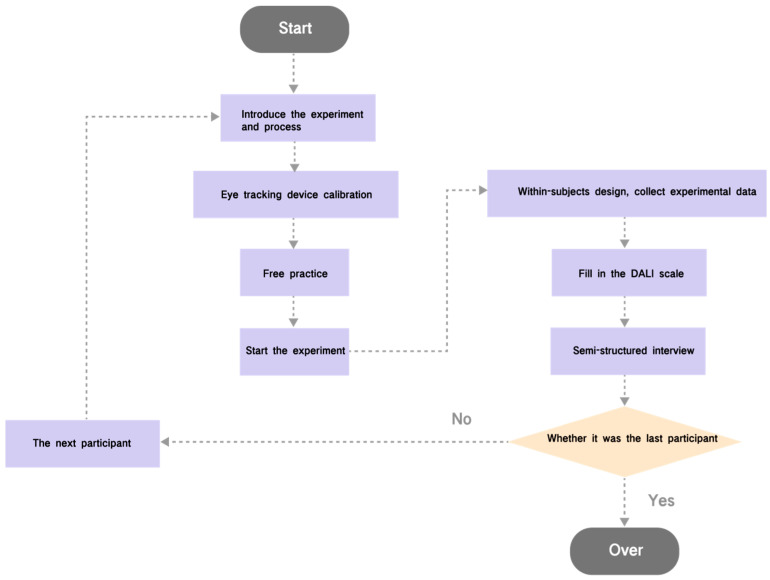
Experimental procedure.

The experimental procedure is shown in Figure 8. Firstly, the purpose, steps, and precautions of the experiment were introduced to the participants, and the design concept, main contents, and the meaning of each visual symbol of the AR-HUD warning ecological interface were introduced. Also, the principle of the AR-HUD warning common interface was introduced to ensure that the participants had a basic familiarity with the two AR-HUD interfaces. Participants were then invited to sit in the driving simulator and wear the eye-tracking device to complete the calibration.

To familiarize themselves with the experimental environment, participants performed a 5-min free practice session, during which they had to manually drive and familiarize themselves with their surroundings. The road setup in this stage was the same as in the formal experiment but without any events. In the formal experiment, a set risk scenario, such as a forward collision, was presented when participants drove into the road segment of a scenario. The subjects were required to detect the danger through the warning interface and press the button on the steering wheel as soon as possible, and then the participant needed to control the vehicle in the horizontal and vertical directions. The three time points of the risk response time (risk situation emergence, risk perception detection, and button press) were automatically recorded by the system. Since participants are required to complete 10 tasks (5 scenarios in 2 conditions), to minimize the impact of practice effect on the experimental results, we used a Latin square design to counterbalance the order of the scenarios. The 5 scenarios appeared in different orders across participants, and each scenario was tested within the schemes of HMI1 and HMI2 alternately. After each experiment, participants were asked to fill out the DALI Scale for Driver Cognitive Workload (see Appendix A), and after completing all experiments, semi-structured interviews (see Appendix B) were conducted with participants to find out how they felt about the interface and why.

### 3.7. Experimental Data Processing

This study collected all subjective and objective data from the participants to analyze the performance of the two HMI schemes. Firstly, the subjective evaluation of the DALI driving load scale included analysis of the score of two HMI schemes in all scenarios, different scenarios, and different scale dimensions. Secondly, the eye-tracking data of two HMI schemes in different scenarios were analyzed. Finally, an analysis was performed on the risk response time of two HMI schemes in different scenarios. In the analysis, we applied the parametric test method (*t*-test) to the data conforming to a normal distribution. For the data that did not conform to the normal distribution, the non-parametric test method (Wilcoxon signed-rank test) was adopted, which was an effective and robust solution when the data did not conform to the parametric test assumptions and the sample size was relatively small.

## 4. Results

Since the recognition rate of the eye movement data of two participants was less than 85%, and the questionnaire data of one participant was incorrect, we eliminated the incorrect data and only retained the valid data of 22 participants for analysis.

### 4.1. Subjective Cognitive Workload

**Figure 9:**
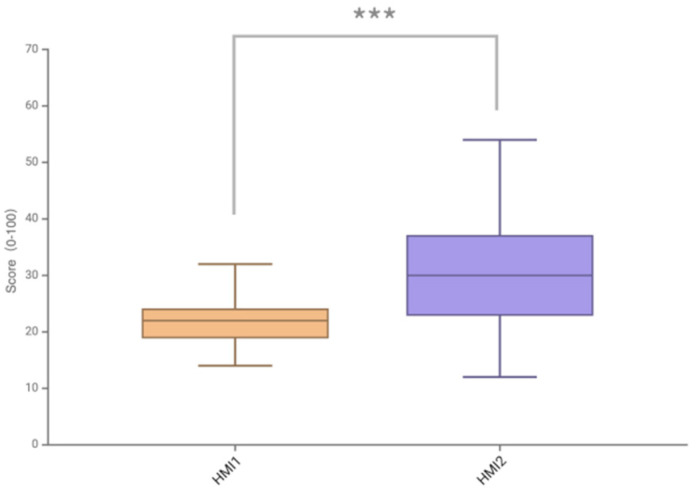
Comparison of DALI total scores of two HMI schemes in all scenarios (*** is *p* < 0.001).

The DALI scale is used to assess the subjective workload of the user, and a lower DALI score indicates a lighter cognitive workload of the participant and a superior interface solution. The comparison of DALI total scores of two different HMI warning schemes in all scenarios shows that the two HMI schemes have significantly different overall scores, as shown in Figure 9. The Shapiro–Wilk test was used a as normality test, and the results showed that the scores of the HMI2 protocol were not normally distributed. Therefore, the non-parametric Wilcoxon signed-rank test was used as an alternative method, and the difference was significant (*p* < 0.001). It can be concluded that the subjective cognitive workload of the AR-HUD warning ecological interface is significantly lower than that of the AR-HUD warning common interface.

**Figure 10:**
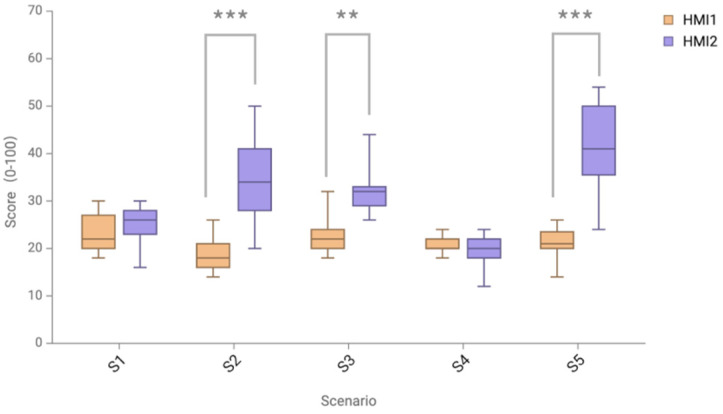
Comparison of DALI total scores of two HMI schemes in different scenarios (*** is *p* < 0.001; ** is *p* < 0.01).

The normality test and paired *t*-test were performed on the score differences of the two HMI schemes in five different scenarios, as shown in Figure 10 and Table 4. The scores in all scenarios passed the normality test, and the difference between the scores of the two HMIs in the S1 and S4 scenarios was not statistically significant (S1, *p* = 0.324; S4, *p* = 0.391). In the S2 scenario, HMI1 (M = 18.91, SD = 3.94) and HMI2 (M = 34.18, SD = 9.18) were significantly different (*p* < 0.001). In the S3 scenario, HMI1 (M = 22.73, SD = 4.13) and HMI2 (M = 32.18, SD = 5.55) were significantly different (*p* < 0.01). In the S5 scenario, HMI1 (M = 21.27, SD = 3.82) and HMI2 (M = 43.09, SD = 8.36) were significantly different (*p* < 0.001). The analysis shows that the subjective cognitive workload of the AR-HUD warning ecological interface is significantly lower than that of the AR-HUD warning common interface in the S2 speed limit warning scenario, S3 lateral collision scenario, and S5 complex scenario.

**Figure 11:**
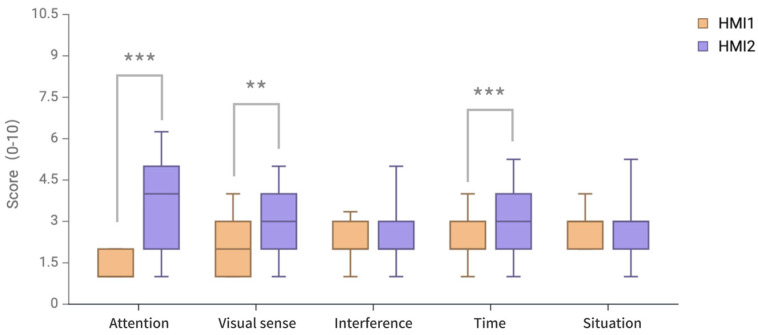
Comparison of DALI score of two HMI schemes in five dimensions (*** is *p* < 0.001; ** is *p* < 0.01).

Data analysis was performed on the scores of different DALI dimensions to examine the sources of cognitive load in different HMI interfaces to verify the strategy’s effectiveness, and the overall dimension scores are shown in Figure 11 and Table 5.

Since most of the dimension data did not conform to a normal distribution for the two schemes (*p* < 0.05), the non-parametric paired difference test method of the Wilcoxon signed-rank test was used, and there was no significant difference between the two HMI schemes in the interference dimension and situation dimension (*p* > 0.05). However, there were significant differences in attention dimension, visual dimension, and time dimension. In the attention dimension, HMI1 (M = 1.39, SD = 0.40) and HMI2 (M = 3.82, SD = 2.97) were significantly different (*p* < 0.001). In the visual dimension, there was a significant difference (*p* < 0.01) between HMI1 (M = 2.44, SD = 0.40) and HMI2 (M = 3.82, SD = 2.97). In the time dimension, there was a significant difference (*p* < 0.001) between HMI1 (M = 2.18, SD = 0.89) and HMI2 (M = 3.16, SD = 1.69).

### 4.2. Objective Cognitive Workload

The objective cognitive workload was evaluated by the average fixation duration and the average pupil diameter in the eye-tracking data.

#### 4.2.1. Average Fixation Duration (AFD)

**Figure 12:**
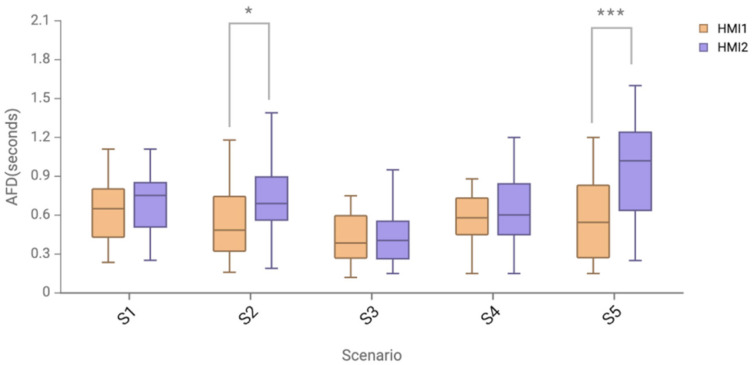
Comparison of average fixation duration of two HMI schemes in different scenarios (*** is *p* < 0.001; * is *p* < 0.05).

The comparison of the average fixation duration of the two HMI schemes in different scenarios is shown in Figure 12 and Table 6. The data in scenarios S1, S2, and S4 conformed to a normal distribution, and the paired *t*-test was used to conclude that there was no significant difference between the two HMI schemes in scenarios S1 and S4 (*p* > 0.05). In the S2 scenario, there was a significant difference (*p* < 0.05) between HMI1 (M = 0.55, SD = 0.30) and HMI2 (M = 0.77, SD = 0.33). Since the data in scenarios S3 and S5 did not conform to the normal distribution, a non-parametric paired difference test was performed using the Wilcoxon signed-rank test, which concluded that there was no significant difference between the two HMI schemes in the S3 scenario (*p* > 0.05). In the S5 scenario, there was a significant difference (*p* < 0.001) between HMI1 (M = 0.59, SD = 0.37) and HMI2 (M = 0.92, SD = 0.44).

#### 4.2.2. Average Pupil Diameter (APD)

**Figure 13:**
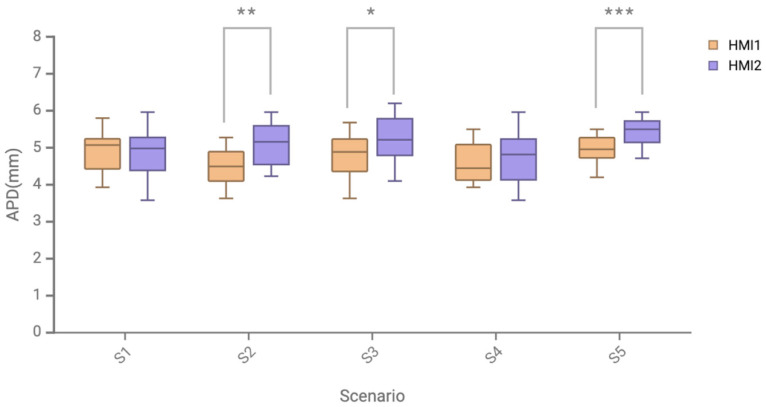
Comparison of average pupil diameter of two HMI schemes in different scenarios (*** is *p* < 0.001; ** is *p* <0.01; * is *p* < 0.05).

The comparison of the average pupil diameter of the two HMI schemes in different scenarios is shown in Figure 13 and Table 7. The data in S1 and S4 failed the normality test (*p* < 0.05), so the Wilcoxon signed-rank test was used for the non-parametric paired difference test, and it was concluded that there was no significant difference between the HMI schemes of the two scenarios. The data in S2, S3, and S5 scenarios had a normal distribution. Using a paired *t*-test, the analysis showed that there was a significant difference between HMI1 (M = 4.50, SD = 0.55) and HMI2 (M = 5.11, SD = 0.58) in S2 (*p* < 0.01); HMI1 (M = 4.80, SD = 0.53) was significantly different (*p* < 0.05) from HMI2 (M = 5.26, SD = 0.61) in S3; HMI1 (M = 4.96, SD = 0.37) was significantly different (*p* < 0.001) from HMI2 (M = 5.44, SD = 0.39) in S5.

### 4.3. Risk Response Time

#### 4.3.1. Risk Perception Time

In the Tobii Pro Lab system, the dynamic AOI is drawn according to the appearance time and location of the warning HMI. The moment when the AOI is drawn is defined as the appearance of the warning HMI, and the moment when the participant first enters the AOI recorded by the Tobii Pro Lab system is defined as the moment when the participant detects the warning HMI. Then, the perception time is the moment when participants detect HMI minus the moment when HMI appears. The specific data of the risk perception time of two HMI schemes are shown in Table 8 and Table 9.

**Figure 14:**
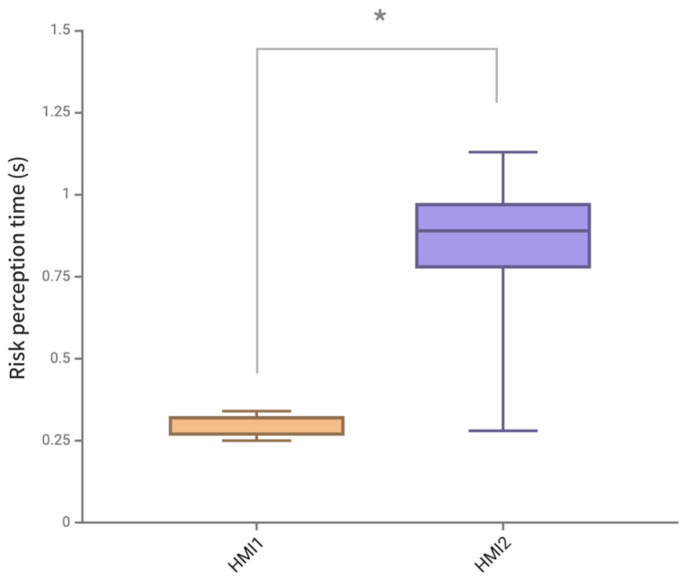
Comparison of the average risk perception time of two HMI schemes in all scenarios (* is *p* < 0.05).

**Figure 15:**
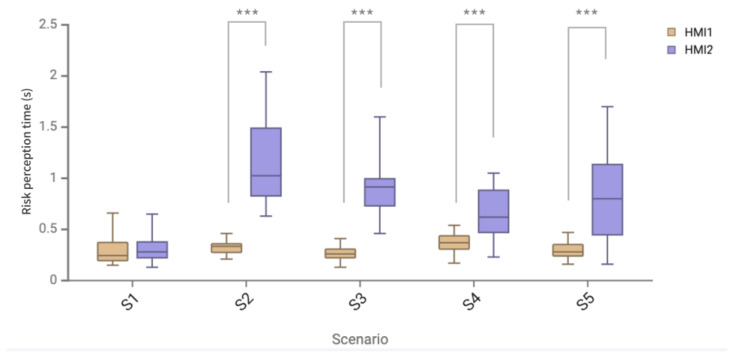
Comparison of average risk perception time of two HMI schemes in different scenarios (*** is *p* < 0.001).

As shown in Figure 14 and Figure 15, the average risk perception time of two HMI schemes was in line with normal distribution, and the paired *t*-test results showed a significant difference in the average risk perception time of two HMI schemes (*p* = 0.022 < 0.05). The analysis of the data results showed that the average perception time of the AR-HUD warning ecological interface in the full warning scenario was significantly shorter than that of the AR-HUD warning common interface by 62.96%, and the standard deviation was reduced by 87.5%. For the average risk perception time data of different scenarios of HMI, the normal test and paired *t*-test showed that there was no significant difference in the S1 scenario, and there were significant differences in S2, S3, S4, and S5 scenarios (*p* < 0.001).

#### 4.3.2. Risk Decision Time

The risk decision time in this experiment is defined as the time from when a participant detects the warning information to when they react. Among them, the time when the participant presses the button is defined as the participant’s reaction moment, and it was explained to the participant that it should be pressed after understanding what driving control behavior is needed. The specific data of the average risk decision time of two HMI schemes are shown in Table 10 and Table 11.

**Figure 16:**
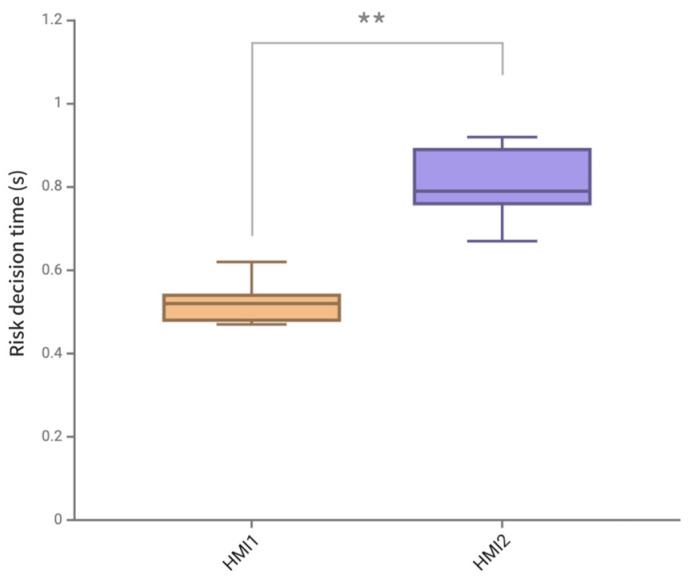
Comparison of average risk decision time of two HMI schemes in all scenarios (** is *p* < 0.01).

As shown in Figure 16, the average risk decision time data of two HMI schemes passed the normality test, and the paired *t*-test results showed that there was a significant difference in the average decision time of two HMI schemes (*p* = 0.003 < 0.01). HMI1 has a significantly lower average risk decision time and a significantly higher bin height interquartile range than HMI2. The analysis of the data results showed that the average decision time of the AR-HUD warning ecological interface in the full scenario was 34.57% shorter than that of the AR-HUD warning common interface, and the standard deviation was reduced by 40%.

### 4.4. Post-Experimental Interview

In the semi-structured interview after the experiment, most participants said that the AR-HUD warning ecological interface can quickly attract their attention when the warning situation occurs, the color change in the graded warning can inform about the danger, and the contrast between the danger and safety zone allows them to understand the speed range that is needed. Compared with HMI2, such clear rules provide them with a greater sense of safety in driving, as well as control over dangerous traffic incidents and smoother operation. However, a small number of participants reported that when the difference between current speed and the safety range is small, it may sometimes be difficult to pay attention to warnings beyond the partial warning. Some participants with more driving experience raised doubts about the interference of the interface because they prefer to judge the deceleration or turning time according to their own experience during the driving process, and the large warning visual area occupies attention resources to a certain extent. In addition, some participants mentioned that when they are not familiar with the AR-HUD warning ecological interface presentation rules, and it may also cause a large cognitive burden on driving at the beginning, but after they become familiar with the interface rules, the adaptability will quickly improve and produce a certain dependence.

### 4.5. Summary of Ecological Interface Cognitive Balanced Design Strategy

Through a comparative experiment, the results show that the design of the ecological interface for the AR-HUD-based driving warning system outperforms the mainstream AR-HUD warning interface in terms of subjective cognitive workload, objective cognitive workload, risk response time, and post-experimental interview. Based on the four comprehensive results, the feasibility of the ecological interface cognitive balance design strategies and models of the AR-HUD-based driving warning system can be verified.

## 5. Discussion

### 5.1. Discussion of Experimental Results

The experimental results support the hypotheses. Hypothesis 1: The AR-HUD warning ecological interface can reduce the driver’s cognitive workload in different single-warning scenarios; Hypothesis 2: In complex multi-warning scenarios, the AR-HUD warning ecological interface can also reduce the driver’s cognitive workload; Hypothesis 3: In both single-warning and multi-warning scenarios, the AR-HUD warning ecological interface can reduce the driver’s risk perception and decision time.

#### 5.1.1. Subjective Evaluation of Cognitive Workload

In the overall evaluation of the DALI subjective scale, the overall cognitive workload of the AR-HUD warning ecological interface is significantly reduced, and the cognitive workload is significantly reduced in speed limit warning, lateral collision warning, and complex multi-warning scenarios. By combining HMI type and scenario cognitive load measurement analysis, in the S2 speed limit warning, S3 lateral collision scenario, and S5 complex multi-warning scenario, the cognitive workload of the AR-HUD warning ecological interface is significantly different from that of mainstream the AR-HUD warning interface, which is also verified by the average pupil diameter results of objective data. In the S2 speed limit warning, S3 lateral collision scenario, and S5 complex multi-warning scenario, the average pupil diameter of the AR-HUD warning ecological interface is significantly lower than that of the mainstream AR-HUD warning interface. Especially in complex multi-warning scenarios, the significant difference of both subjective and objective cognitive load data is *** *p* < 0.001, indicating that this warning interface has a particularly significant effect on reducing cognitive load in complex-warning scenarios. This shows the effectiveness of the cognitive load adjustment method based on the distraction effect. Integrating it into one body and presenting different warning interfaces reduces the situation of distraction compared to presenting multiple warning information in complex scenarios so that drivers can quickly understand the current scenario information and make responses. In addition, in the collision warning scenario, the cognitive load score of the AR-HUD warning ecological interface in the S3 horizontal collision scenario is better, and in the complex-warning scenario, combined with the horizontal collision scenario, it also shows a better cognitive load score, which can confirm the effectiveness of the AR-HUD warning ecological interface in displaying side obstacles. The obstacle direction guide of the side obstacle and the suggested speed control display can better guide the driver to pay attention to the obstacle in time and quickly understand the operation suggestion for avoiding the obstacle.

DALI scale dimension evaluation data show that in the three dimensions of attention demand, visual demand, and time demand, the AR-HUD warning ecological interface has significantly better scores than HMI2. Attention demand indicates the effort to find the warning information in the visual field. The score with low effort indicates the effectiveness of the strategy of presenting the AR-HUD warning ecological interface in the visual fovea of the AR-HUD. Moreover, HMI2 does not significantly increase compared with the average fixation duration in the eye-tracking data. This indicates that the warning interface will not occupy the driver’s attention resources too much and may guide the driver to look in the direction of obstacles. In terms of visual demand, the AR-HUD warning ecological interface has significantly lower visual requirements than that of HMI2. In other words, more visual processing is not required when processing the warning interface provided by the AR-HUD, indicating the effectiveness of the fusion strategy of SBB, RBB, and KBB. Compared with the warning interface that only provides one SRK classification, the existence of multiple levels of information in the AR-HUD warning ecological interface enables the driver to better understand the environmental information. A better time demand score indicates that it takes less time for the driver to understand the information in the warning interface to perform the driving operation. This indicates the effectiveness of SBB-based information, which enables the perception and action to be coupled and allows skill-based responses to the current situation without much cognitive control. Moreover, because the information of RBB and KBB is integrated into the interface, while the operation is being carried out, the driver is also able to understand the source and context of the danger. In other dimensions, the interference dimension and situational pressure dimension are not significantly different from the AR-HUD warning common interface, indicating that the AR-HUD warning ecological interface does not cause excessive interference and pressure for the driver while attracting the driver’s attention and improving the driver’s speed of understanding information. In addition, it also shows that the visual fovea area is suitable for the presentation of the warning interface, because when a danger occurs, the importance of solving the danger rises to the top of the importance level of the current driving task, and warning information and corresponding action suggestions are the most urgent information for the driver, so it will not bring too much interference and situational pressure to the driver.

#### 5.1.2. Cognitive Load Eye-Tracking Data

In the data results of average fixation duration, the average fixation duration of the AR-HUD ecological interface in the S2 speed limit warning scenario and the S5 complex-warning scenario is significantly shorter than that of the AR-HUD warning common interface, indicating that, in these two warning scenarios, the AR-HUD warning ecological interface keeps the driver’s attention more in the scenario and the control of dangerous situations and reduces the cognitive load on understanding. In the speed limit warning scenario without dangerous sources, the average fixation duration of the AR-HUD warning ecological interface is shorter, which may be because the warning ecological interface is presented in the driver’s visual fovea by the way of 3D registration, compared with the AR-HUD warning common interface which is presented in the focal length field at the lower part of the visual center by the way of registration. The driver can avoid the switch of visual focal length so that the overspeed warning information can be received more intuitively and safely. This may be because the warning ecological interface allows the driver to realize speed control only by using the peripheral part of the non-visual focus when speeding, without paying attention to changes in the specific speed value, which can reduce the cognitive load of the driver for the warning information and allow them to pay more attention to the road and the main driving task.

In the data results of average pupil diameter, in the S2 speed limit warning, S3 lateral collision scenario, and S5 complex multi-warning scenario, the average pupil diameter of the AR-HUD warning ecological interface is significantly smaller than that of the mainstream AR-HUD warning common interface. Especially in complex multi-warning scenarios, the significant difference between subjective and objective cognitive load data is *p* < 0.001, indicating that this warning interface has a particularly significant effect on reducing cognitive load in complex-warning scenarios. Combined with subjective and objective cognitive load measurement data, the research results show that, compared with the mainstream AR-HUD, the AR-HUD warning ecological interface design can effectively reduce cognitive workload and has significant interface advantages in the S2 speed limit warning, S3 lateral collision scenario, and S5 complex multi-warning scenario. The results show that the AR-HUD warning ecological interface design has the potential to reduce drivers’ cognitive load in traffic control scenarios, obstacle collision scenarios, and warning scenarios involving drivers’ horizontal and vertical control. The versatility of the warning interface has also been well verified in complex scenarios, indicating its applicability in a realistic complex traffic safety environment, and complex scenarios involve complex operations that require drivers to decelerate and turn back vertically and horizontally at the same time. The cognitive load data show that the ecological interface has the potential to reduce the cognitive load of drivers to a large extent in complex traffic environments. Based on providing sufficient information for the driver, the driver can quickly understand and make appropriate and reasonable responses to improve safety.

#### 5.1.3. Risk Response Time

According to the analysis of risk perception time data, the average perception time of the AR-HUD warning ecological interface in a full warning scenario is significantly shortened by 62.96% compared with that of the AR-HUD warning common interface, and the standard deviation is reduced by 87.5%. This significant difference also shows that the presentation of warning information in the visual fovea can improve the perception speed. Compared with the AR-HUD warning common interface, the driver does not need to search for warning information in the world coordinates but can quickly find the position in the center of the field of vision. In the forward collision warning scenario of S1, there is no significant difference in the danger perception time between the ecological interface and the common interface of AR-HUD warning, which also confirms this view from the side, because, in S1, both interfaces are presented in the position of the visual fovea. In addition, the large standard deviation difference also indicates that the AR-HUD warning ecological interface can make the driver maintain a faster perception time in different warning scenarios, and the effect of reducing the risk perception time is stable.

The analysis of risk decision time data shows that the average decision time of the AR-HUD warning ecological interface in the whole scenario is shortened by 34.57%, and the standard deviation is reduced by 40% compared with the AR-HUD warning common interface. There is also a significant difference in the risk decision time, indicating the effectiveness of the ecological interface design strategy. Combining the information of the three classification methods of SRK, the driver can stimulate skill-based behavior, respond quickly, and understand the current situation through RBB and KBB at a more abstract level to make deterministic decision operations.

### 5.2. Limitations and Future Work

Although this study has made some achievements regarding the topic of focus, there are still some theoretical hypotheses that have not been empirically tested. In particular, as mentioned in the design framework, personalized adjustment and display of information can be carried out according to SBB, RBB, and KBB for drivers with different driving experience levels. The potential utility of this personalized information display based on the reversal effect of expertise in improving drivers’ cognitive fitness deserves further exploration.

In addition, the participants recruited in this study were young and lacked a broad age range. In future work, we will focus on other age groups such as the elderly to improve the wider applicability and validation of this study.

Additionally, this study presents five scenarios, and the impact of different scenarios on the dependent variables is also a topic worth exploring. However, since this study focuses on verifying the feasibility of the cognitive balance ecological interface design framework for the AR-HUD driving warning system, using a comparative experiment between two schemes of HMIs, it does not focus on the influence of different scenarios. In future research, the sample size can be increased, and more targeted experiments can be redesigned to explore this issue in more detail.

Finally, the role of the warning interface of threshold visualization in ecological interface design in improving driving performance, including whether it can promote more accurate steering and more stable speed control of drivers, is also worth further research. These research topics not only have theoretical value but also have important significance in practical application.

## 6. Conclusions

In conclusion, this study analyzes the impact of the AR-HUD driving warning system interface on drivers’ cognitive load and aims to build a cognitively balanced AR-HUD driving warning interface based on ecological interface design theory. By reducing the interface cognitive load and improving the driver’s operational understanding and response efficiency, it is committed to reducing the traffic accident rate and enhancing driving safety. Through literature review and case analysis, we discuss the sources of cognitive load of the mainstream AR-HUD interface in different warning situations. By combining cognitive load regulation methods with the dimensions of AR-HUD interface design, and adopting the principle of ecological interface design, the design strategy and framework of cognitive balanced ecological interface for the AR-HUD driving warning system are proposed. Through experimental verification, it is proved that these strategies can effectively reduce cognitive load in single- and complex-warning scenarios, which is better than the existing mainstream AR-HUD warning interface design. Therefore, the results of this study provide theoretical and practical support for improving the practicability and efficiency of the AR-HUD driving warning system. The design strategy and framework of the cognitive balanced ecological interface we proposed have reference value for research in the same field and help to promote a wider range of application scenarios.

## Figures and Tables

**Table 1:** Basis of theoretical analysis.

Number	A	B	C	D
Name	Principle and constitution of DWS	Features of AR-HUD-based driving warning system	Cognitive load theory (CLT)	Ecological interface design (EID)
Content	(1) Principle (human–machine interface layer; decision-making layer; sensing layer)	(1) Revelation principle	(1) Theoretical basis	(1) Theoretical basis
	(2) System structure and classification	(2) Interface case studies (5 mainstream and 2 manufacturers)	(2) Type and balance principle	(2) Abstract hierarchy analysis
	(3) Hierarchical warning mechanism	(3) Warning interface design features	(3) Driving cognitive load (DCL)	(3) SRK classification framework and semantic mapping

**Table 2:** Basic information of participants.

Information	Participants Data
Gender	12 (male), 13 (female)
Age	22–27 (mean = 25.36, SD = 1.82)
Driving experience	5 (1–2 years), 3 (2–3 years), 5 (4–5 years), 12 (5+ years)
Average kilometers per week	13 (100–200 km), 6 (200–300 km), 3 (300–400 km), 3 (500+ km)

**Table 3:** Dependent variable quantitative data extraction periods.

Metric		Data Extraction Periods
Driving Activity Load Index	DALI scale	After each scenario experiment (the location of red dots in Figure 7)
Eye-Tracking Data	Average Fixation Duration (AFD)	Each scenario experiment officially begins–ends (areas with purple dots in Figure 7)
	Average Pupil Diameter (APD)	
Risk Response Time (the area with yellow dots in Figure 7)	Risk Perception Time	The time difference between the occurrence of a risk and the participant’s perception of the risk in each scenario
	Risk Decision Time	In each scenario, participants provided feedback after identifying a risk

**Table 4:** Statistics of average DALI total score of two HMI schemes in different scenarios.

	S1	S2	S3	S4	S5	Mean	SD
HMI1	23.64	18.91	22.73	20.73	21.00	21.45	1.84
HMI2	25.09	34.18	32.18	19.64	41.50	30.84	8.43

**Table 5:** Statistics of average DALI score of two HMI schemes in different dimensions.

	Attention	Visual Sense	Interference	Time	Situation	Mean	SD
HMI1	1.44	2.15	2.20	2.18	2.76	2.15	0.47
HMI2	3.82	2.89	2.55	3.16	3.00	3.08	0.47

**Table 6:** Statistics of average fixation duration (unit: seconds) of two HMI schemes in different scenarios.

	S1	S2	S3	S4	S5	Mean	SD
HMI1	0.63	0.55	0.43	0.54	0.59	0.55	0.07
HMI2	0.70	0.77	0.43	0.63	0.92	0.69	0.18

**Table 7:** Statistics of average pupil diameter (unit: millimeters) of two HMI schemes in different scenarios.

	S1	S2	S3	S4	S5	Mean	SD
HMI1	4.86	4.50	4.81	4.61	4.95	4.75	0.19
HMI2	4.92	5.11	5.26	4.75	5.44	5.10	0.27

**Table 8:** Average risk perception time (unit: seconds) of two HMI schemes in different scenarios.

	Time of Risk Occurrence		Risk Time is Detected		Perception Time	
	HMI1	HMI2	HMI1	HMI2	HMI1	HMI2
S1	10.58	11.23	10.85	11.47	0.27	0.28
S2	13.75	13.89	14.07	15.02	0.32	1.13
S3	11.45	12.78	11.70	13.75	0.25	0.97
S4	14.52	13.78	14.86	14.45	0.34	0.78
S5	11.34	15.49	11.66	16.38	0.32	0.89

**Table 9:** Statistics of average risk perception time (unit: seconds) of two HMI schemes in all scenarios.

	Mean	SD	Minimum	Maximum	Median
HMI1	0.30	0.04	0.25	0.34	0.32
HMI2	0.81	0.32	0.28	1.13	0.89

**Table 10:** Average risk decision time of two HMI schemes in different scenarios (unit: seconds).

	Time Risk Is Detected		Moment of Reaction		Reaction Time	
	HMI1	HMI2	HMI1	HMI2	HMI1	HMI2
S1	10.85	11.47	11.39	12.23	0.54	0.76
S2	14.07	15.02	14.54	15.91	0.47	0.89
S3	11.70	13.75	12.22	14.42	0.52	0.67
S4	14.86	14.45	15.34	15.24	0.48	0.79
S5	11.66	16.38	12.28	17.30	0.62	0.92

**Table 11:** Statistics of average risk decision time of two HMI schemes in different scenarios (unit: seconds).

	Mean	SD	Minimum	Maximum	Median
HMI1	0.53	0.06	0.47	0.62	0.52
HMI2	0.81	0.10	0.67	0.92	0.79

## Data Availability

The raw data supporting the conclusions of this article will be made available by the authors on request.

## Supplementary Materials

The following supporting information can be downloaded at: https://www.mdpi.com/article/10.3390/s24248010/s1, Note S1: Description of variable names in the design framework; Note S2: Prototype design. References [32,54] are cited in the supplementary materials.

## Appendix A

**Table A1:** Driving Cognitive Workload DALI Scale.

Number	Content	Question Types	Scale
1	Subject number (this question is filled in by the experimenter)	Fill in the blanks	-
2	Experiment scenario (this question is filled in by the experimenter)	Single-choice question	1–5 (scenario)
3	Experiment interface (this question is filled in by the experimenter)		1–2 (HMI)
4	Attention need		1–10 (score)
5	Visual need		
6	Disturb		
7	Time need		
8	Situational pressure		

## Appendix B

Semi-structured interview outline.

Opening of the interview:

(1) State the purpose and expected time of the interview.
(2) Emphasize privacy protection and ensure that respondents can freely express their views.

Text of the interview:

1. User experience of AR-HUD warning ecological interface.
  (1) Describe how you feel when using the AR-HUD warning ecosystem interface.
  (2) During use, do you think the information displayed on the interface is clear and easy to understand?
  (3) Have you encountered any difficulties in using the AR-HUD warning ecosystem interface?
2. Cognitive load assessment.
  (1) Do you feel an increased cognitive burden when using the AR-HUD warning ecological interface while driving?
  (2) How do you think the AR-HUD warning ecosystem interface affects your attention allocation?
  (3) When using the interface, do you need to spend extra effort to understand the warning information?
3. Safety and trust.
  (1) Does the use of an AR-HUD warning ecological interface enhance your driving security?
  (2) What is your assessment of the accuracy and reliability of the AR-HUD warning ecosystem?
  (3) Under what circumstances do you particularly trust or doubt the alerts of the warning system?
4. Personalize your needs and suggestions.
  (1) In what ways do you think the AR-HUD warning ecological interface can be further improved?
  (2) Do you have any personalized needs or preferences for the display of warning information?
  (3) What new features or information would you like to add in a future release?

End of interview:

(1) Summarize the main points of the interview and ask the interviewees if there is anything else they would like to add.
(2) Express gratitude for the interviewee’s time and contribution.
(3) Explain the subsequent research progress and how to obtain the research results.
