# Supplementary material for: Design and Evaluation of Ecological Interface of Driving Warning System Based on AR-HUD
Source: Sensors (Basel). 2024 Dec 15;24(24):8010. doi: 10.3390/s24248010 (PMC11679450; doi:10.3390/s24248010)
Supplement: Supplementary file 1 [file sensors-24-08010-s001.zip › sensors-3288012-supplementary.pdf]

SUPPLEMENTARY MATERIAL

Design and evaluation of ecological interface of driving warning system based on AR-HUD

Jun Ma [1,2], Yuhui Li [1], Yuanyang Zuo [1*]

1 College of Design and Innovation, Tongji University, Shanghai, China; majun.tongji@foxmail.com (J.M.); yottoli@foxmail.com (Y.L.)

2 School of Automotive Studies, Tongji University, Shanghai, China;

* Correspondence: zuoyy@tongji.edu.cn (Y.Z.)

Note S1: Description of variable names in the design framework.

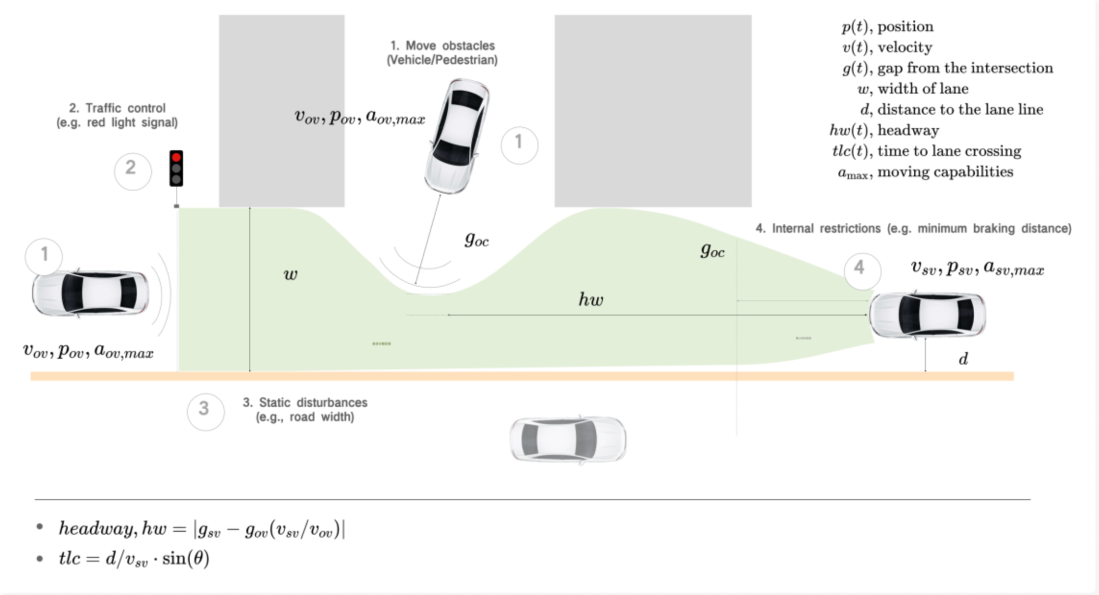

Figure S1. Safe Driving Area (adapted from Gibson [S1]).

Critical warning threshold: $HW*$ (headway)

As shown in Figure S1, the real-time headway value $hw(t)$ of the vehicle can be calculated according to the current real-time position and driving speed between the two vehicles. The specific formula is as follows:

$$hw(t) = \left| g_{sv}(t) - g_{ov}(t)\left(\frac{v_{sv}(t)}{v_{ov}(t)}\right) \right|. \tag{1}$$

Safe distance threshold: S_{safe}

Based on the set $HW*$, without considering the steering angle of the vehicle, the overall safe driving zone can be basically expressed by the spatial distance S_{safe} from the limit, and the calculation formula is shown in equation (2)

$$S_{\text{safe}} = v \times HW* + \frac{v^2}{2 \times a_{\text{decel}}} + s_{\text{buffer}}. \tag{2}$$

Here, v is the vehicle speed, $HW*$ can be equivalent to the reaction time reserved for the driver, but also the warning threshold, the common value is 1 to 2.5 seconds; a_{decel} is the deceleration of the vehicle, depending on the braking system and road conditions of the vehicle, when a_{decel}=a_{max} (maximum deceleration of the vehicle), that is, the minimum braking distance; s_{buffer} is an additional safety buffer distance, which may vary according to the driving

environment, usually ranging from a few meters to tens of meters.

In driver assistance systems, these parameters are usually programmed into the system, which dynamically calculates S_{safe} based on real-time data.

Sometimes, the safe distance threshold is set more conservatively to ensure that the vehicle can stop safely under a variety of conditions. The system designer or engineer determines an appropriate S_{safe}threshold based on the specific application scenario, vehicle characteristics, expected operating environment (urban roads, highways, etc.), and applicable regulations or guidelines.

Main types of traffic restrictions:

Specific types of traffic restrictions, as shown in Figure S1, in the traffic scenario that restricts the safe driving area, there are four main types of restrictions or obstacles:

1. Move obstacles.

Other road roles at the physical functional level, such as vehicles or pedestrians, include obstacles that are currently stationary but have the possibility of moving. When the acceleration of both the obstacle and the vehicle changes, the relative acceleration of the obstacle and the vehicle needs to be considered in the safe driving area. Assuming that acceleration varies with time, the speed of the vehicle at any given point in time depends on the acceleration from the initial time to time t:

$$v(t) = v_0 + \int_0^t a(\tau)\, d\tau. \tag{3}$$

Here, v_0 is the initial speed and $a(\tau)$ is the acceleration of the obstacle at time τ, then the safe driving area takes into account the relative acceleration of the vehicle in front and the vehicle itself, as shown in Formula 4:

$$S_{\text{safe}}(t) = v_{\text{initial}} \cdot HW^* + \int_0^T \left(v_{sv}(t) - v_o(t)\right) dt + s_{\text{buffer}}. \tag{4}$$

Here, v_{initial} is the speed of the car at the beginning of braking; $v_{sv}(t)$ is the speed of the subject vehicle in time; $v_o(t)$ is the speed of the obstacle at time t, when the obstacle is stationary, the value is 0; Tis the total time from when the car starts to slow down to stop.

2. Traffic control.

That is, traffic lights and speed limit signs at the physical function level belong to invisible barriers, and only longitudinal speed limits are considered in most scenarios, namely:

$$v(t) = v_0 + \int_0^t a(\tau)\, d\tau \le V_{\text{max}}. \tag{5}$$

When in the speed limit section, the maximum speed limit of the current section; When the traffic light is red, the stopping area at the intersection should be 0 km/h.

3. Static disorder.

Road conditions belonging to the physical function level, such as road width, etc., in such obstacles, the lane deviation is mainly considered. The variable describing the change in lateral lane position is lane departure distance $D(t)$, which represents the lateral distance of the vehicle from the center or boundary of the lane at a given moment. Its formula is as follows:

$$D(t) = L(t) + d(t)\sin\big(\theta(t)\big). \tag{6}$$

Here, $L(t)$ is the lateral position of the vehicle, that is, the distance relative to the center of the lane or the boundary of the lane, $d(t)$ is the distance in the direction of the vehicle, and $\theta(t)$ is the angle between the direction of the vehicle and the direction of the lane.

Generally, the early warning threshold of Lane deviation is calculated through TLC (Time to Lane Crossing, distance from time to lane), as shown in Formula 7:

$$TLC = \frac{-v_{\text{lateral}} + \sqrt{v_{\text{lateral}}^2 - 2 \cdot a_{\text{lateral}} \cdot L(t)}}{a_{\text{lateral}}}. \tag{7}$$

Here, v_{lateral} and a_{lateral} are the lateral speed and acceleration of the vehicle, a_{lateral} is related to the wheelbase of the vehicle and the current steering angle θ. TLC is often used to assess the urgency of departure, and a small TLC value means the vehicle is about to leave the lane and immediate action is needed.

In practice, the TLC is used to decide when to issue a lane departure warning: If the TLC falls below a certain threshold, the system may determine that a warning is needed. The $D(t)$ can be used to trigger the display position of this warning, such as a specific indication on the HUD or dashboard when the vehicle is approaching the lane boundary.

4. Internal restrictions.

What can be classified as the interaction of road conditions and road roles at the physical system level, such as the braking performance of the vehicle, as well as the frictional resistance of the road, all affect the minimum braking distance. Taking into account the internal limitations of the vehicle, this limitation is generally taken into account in the calculation of the safe driving area, combined with the maximum deceleration of the vehicle a_{max} and the setting of a reasonable s_{buffer} value (see Formula 2) for avoidance.

SRK classification of AR-HUD warning interface elements.

SRK classification framework refers to the classification of human behaviors based on skills, rules and knowledge [S2]. Different types of AR-HUD interface information correspond to different SRK classifications: a specific location is associated with knowledge-based behaviors, while a dynamic environment stimulates rules-based behaviors. Advising on actions is directly skill-based behavior, and their correlation is analyzed in detail below:

1. Location presentation: Stimulating Knowledge-based Behavior (KBB).

A specific location is represented by the presentation of coordinates of a specific obstacle or a traffic sign, and the explanation in the interface is low. It only explains the location of the limit, but does not provide suggestions and guidance for driving operations. Therefore, the information elements belonging to the sign category in SRK classification correspond to the stimulation of knowledge-based behaviors. Drivers need to have a higher level of driving schema, that is, they need to carry out more cognitive control -- evaluate the current state of limitations, interpret and analyze them, and finally transform them into driving operations, so as to complete the judgment and processing of the current situation.

2. Describe the dynamic environment: Inspire Rule-based Behavior (RBB).

To present the dynamic environment around the driver, that is, to predict the source of the restriction, to show the future path of the danger source, or to predict the restriction information of the current road, so that the driver can prepare the corresponding driving operation in advance. Within the scope of symbols in the SRK classification, such information can trigger the driver's existing schema-that is, observe the predicted danger, identify the action that can be taken, and finally extract the optimal driving strategy and take action.

3. Suggest actions: Inspire Skill-based Behavior (SBB).

Directly provide the driver with action suggestions, such as stop, slow down or turn, can quickly

let the driver without judgment can immediately execute the corresponding operation, the interface interpretation is strong, can let the driver immediately understand the meaning of the corresponding information. This corresponds to the signal information element in the SRK classification, which can stimulate skill-based behavior, directly contribute to the coupling of perception and action, and is also an early warning mode that requires the least driving schema.

In the interface presentation, three different presentation modes are combined to promote three different driving behaviors (SBB, RBB, KBB), which can enable drivers to make the fastest operational response, and at the same time, they can understand the connection between current environmental information and driving operations, and build relevant driving schemas to better respond to other dangerous traffic incidents.

Note S2: Practice of prototype design of AR-HUD warning interface in a single warning scenario.

1. Forward collision warning scenario prototype design.

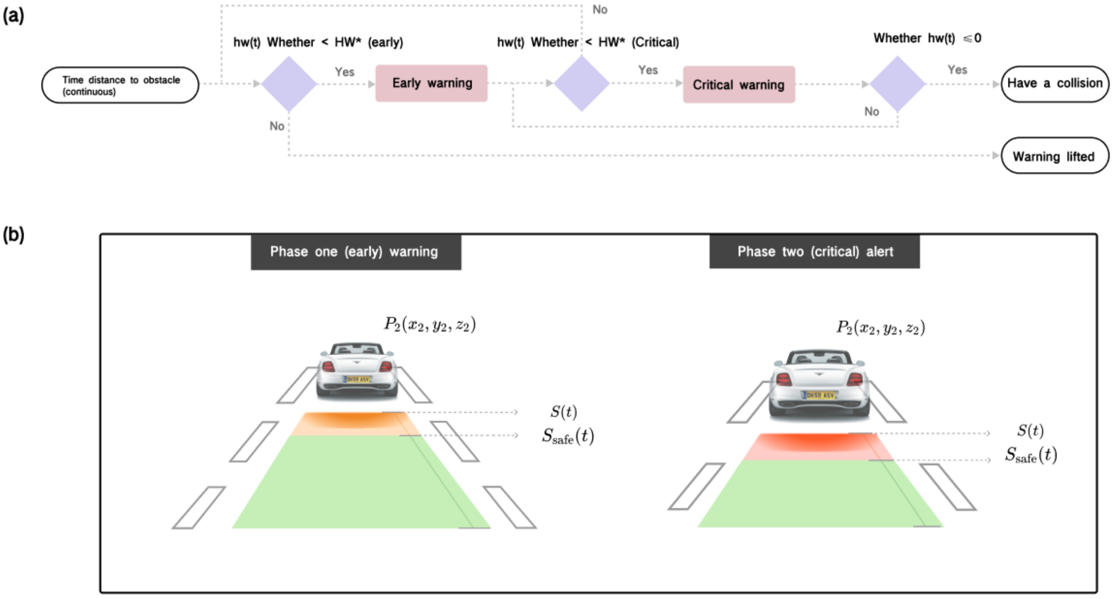

Figure S1. Forward collision warning. (a) Forward collision warning flow chart. (b) Forward collision warning AR-HUD interface design.

The warning flow chart of the forward collision warning system is shown in Fig. S1(a). The warning system monitors the distance between the current and the preceding vehicle in real-time and calculates the distance between the two vehicles according to:

$$hw(t) = \left| g_{sv}(t) - g_{ov}(t)\left(\frac{v_{sv}(t)}{v_{ov}(t)}\right)\right|.$$

Here, $g_{sv}(t)$ is the distance between the subject vehicle and the intersection, $g_{ov}(t)$ is the distance between the obstacle vehicle and the intersection, $v_{sv}(t)$ is the speed of subject vehicle, and $v_{ov}(t)$ is the speed of obstacle vehicle. According to the threshold of headway ($HW*$), if $hw(t) < HW*_{\text{early}}$, the early warning interface is presented; if $hw(t) < HW*_{\text{critical}}$, the critical warning interface is presented. According to the real-time transformation of $hw(t)$, the warning interface can be switched between the early warning interface and the critical warning interface. When $hw(t) > HW*_{\text{early}}$ is detected, the warning will be lifted and the warning interface won't be displayed. Figure S1(b) shows the AR-HUD interface design of forward collision warning. The safe distance $S_{safe}(t)$ is defined as:

$$S_{\text{safe}}(t) = v_{\text{initial}} \cdot HW * + \int_0^T \big(v_{st}(t) - v_0(t)\big)dt + s_{\text{buffer}}.$$

Here, v_{initial} is the speed of the subject vehicle at the beginning of braking, T is the total time from the beginning of braking to stop, s_{buffer} is the extra safety buffer distance ranging from a few meters to tens of meters.

2. Speed limit warning scenario prototype design.

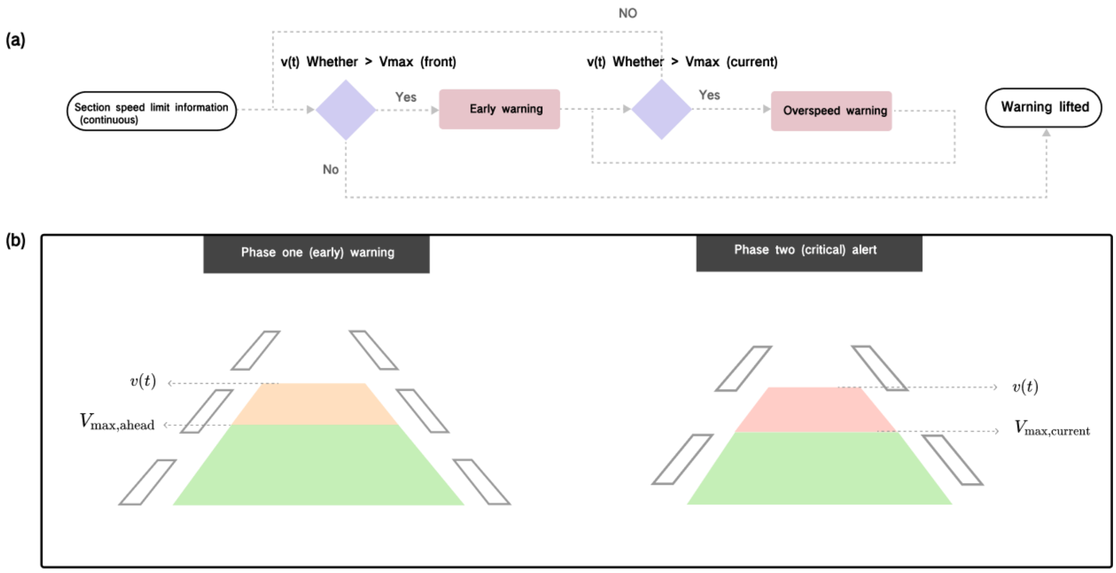

Figure S2. Speed limit warning. (a) Speed limit warning flow chart. (b) Speed limit warning AR-HUD interface design.

The warning flow chart of the speed limit warning system is shown in Fig. S2(a). The speed limit warning system monitors the speed limit information of the front and the current road section in real-time and compares the current speed limit $v(t)$ to the speed limit of the road V_{max}. If $v(t) < V_{\mathrm{max,ahead}}(t)$, the early speed limit warning interface of phase one is presented; if $v(t) < V_{\mathrm{max,current}}(t)$, the warning interface of phase two is presented. The priority of the current speed limit warning $V_{\mathrm{max,current}}(t)$ is higher than that of $V_{\mathrm{max,ahead}}(t)$. When $v(t)$ is detected less than the latter two, the warning will be lifted. Figure S2(b) shows the AR-HUD interface design of speed limit warning.

3. Lateral collision warning scenario prototype design.

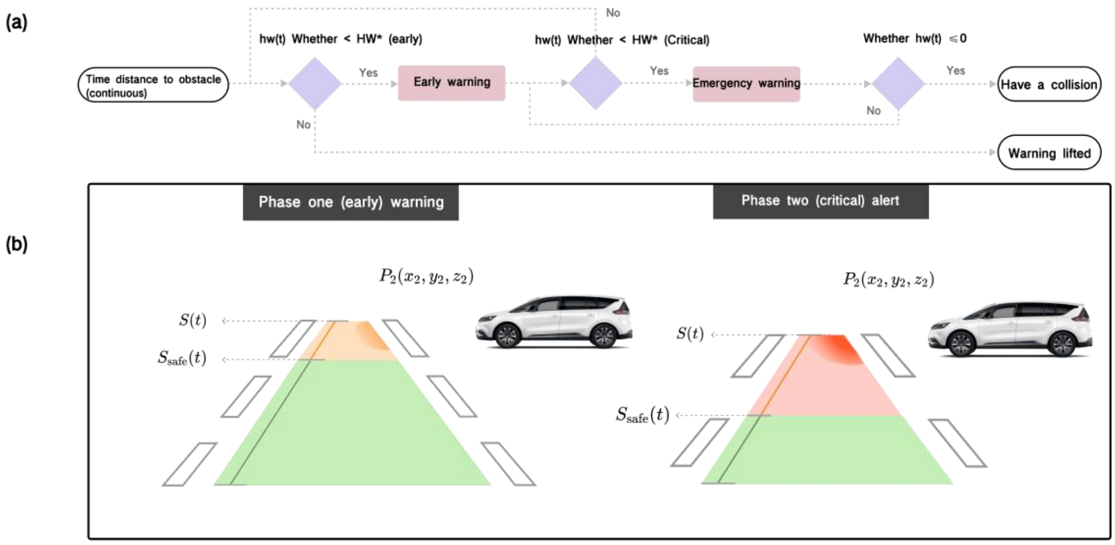

Figure S3. Lateral collision warning. (a) Lateral collision warning flow chart. (b) Lateral collision warning AR-HUD interface design.

The warning flow chart of the lateral collision warning system is shown in Fig. S3(a). The warning display time takes $HW*$ as the threshold. if $hw(t) < HW*_{\mathrm{early}}$, the early warning

interface is presented; if $hw(t) < HW *_{\text{critical}}$, the critical warning interface is presented. Figure S3(b) shows the AR-HUD interface design of speed limit warning.

4. Lane departure warning scenario prototype design.

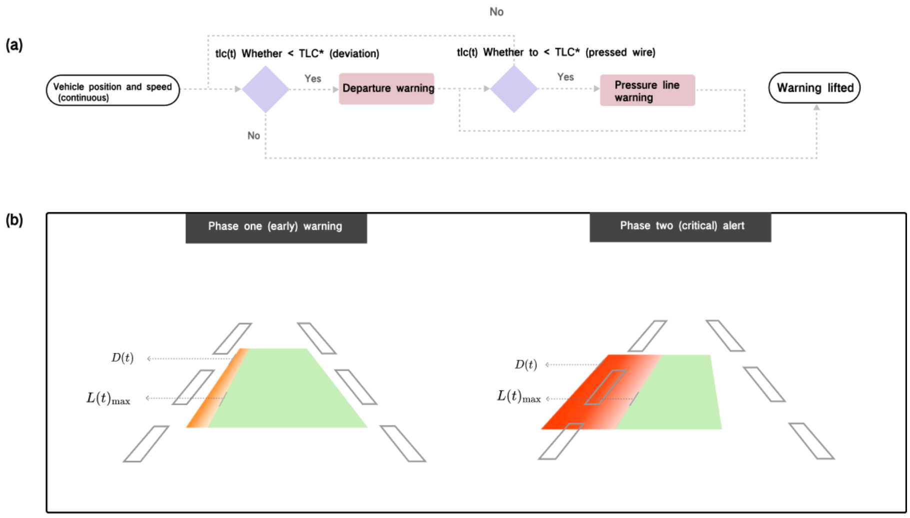

Figure S4. Lane departure warning. (a) Lane departure warning flow chart. (b) Lane departure warning AR-HUD interface design.

The warning flow chart of the lane departure warning system is shown in Fig. S4(a). The lane departure warning is triggered by time to lane crossing ($TLC *$), including lane departure threshold $TLC *_{\text{deviate}}$ and crossing line threshold $TLC *_{\text{cross}}$. When $tlc(t) < TLC *_{\text{deviate}}$, the lane departure warning of phase one is triggered; when $tlc(t) < TLC *_{cross}$, the crossing line warning of phase two is triggered. According to the real-time change of $tlc(t)$, the warning switches between the interface of departure warning and crossing line warning, and when $tlc(t) > TLC *_{\text{deviate}}$, the warning will be lifted. Figure S4(b) shows the AR-HUD interface design of lane departure warning. The safety area is represented by the maximum allowable lateral departure distance $L(t)_{\max}$, and $L(t)$ is associated with TLC as follows:

$$TLC = \frac{-v_{\text{lateral}} + \sqrt{v_{\text{lateral}}^2 - 2 \cdot a_{\text{lateral}} \cdot L(t)}}{a_{\text{lateral}}}.$$

Here, v_{lateral} is the lateral speed of the subject vehicle, a_{lateral} is the lateral acceleration, which is related to the wheelbase of the subject vehicle and the current steering angle θ. When the phase one of lane departure warning $TLC *_{\text{deviate}}$ is triggered, the value of $L(t)$ is $L(t)_{\max}$. $D(t)$ is the lateral lane offset defined as follows:

$$D(t) = D_0 + v_{\text{lateral},0} \cdot t + \int_0^t a_{\text{lateral}}(\tau) d\tau,$$

Which can reflect the current lateral acceleration.

Practice of prototype design of AR-HUD warning interface in complex warning scenario.

In real traffic situations, dangerous scenarios are relatively complex, and multiple warnings may occur at the same time. To detect the cognitive load and availability of the warning interface in complex traffic scenarios, a complex warning scenario based on two warnings is set up, and a more urgent scenario that can cover the horizontal and vertical operations of drivers is selected for prototype design and test.

1. Complex warning scenario design.

In a real traffic scenario, when the driver deviates from the lane and another car comes from the opposite lane at the same time, there is a possibility of collision between the two vehicles. This situation is often seen when the driver tries to occupy the opposite lane to overtake, or when the driver is distracted by driving and unintentionally deviates from the lane, there may be a collision or scratches in the opposite lane. The top view of this traffic scenario is shown in Fig. S5. The subject vehicle first has the risk of lane departure (①), triggering the lane departure warning. Then, the system detects that other vehicles are coming from the opposite lane. If the subject vehicle and the opposite vehicle continue to drive at the current transverse and longitudinal acceleration, there is the possibility of lateral collision (②), which triggers the lateral collision warning.

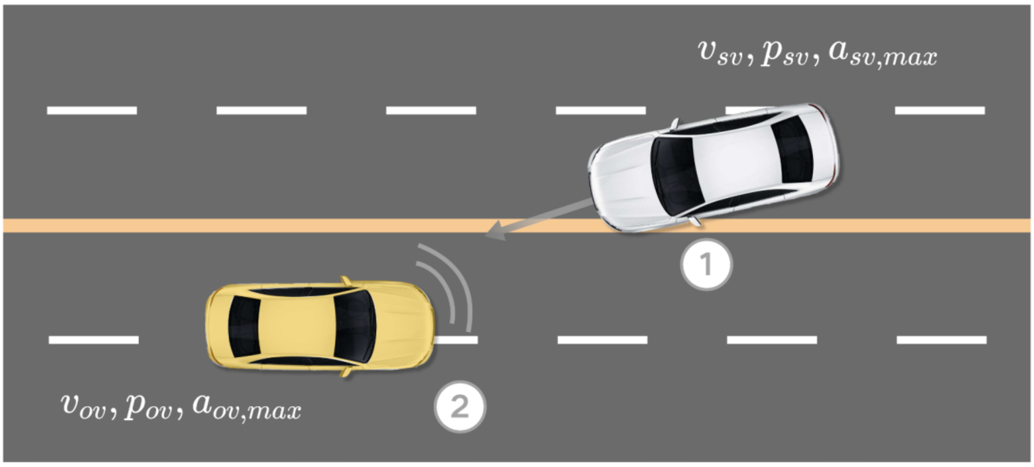

- $headway, hw = |g_{sv} - g_{ov}(v_{sv}/v_{ov})|$
- $tlc = d/v_{sv} \cdot \sin(\theta)$

Figure S5. Complex scenario design: lane departure and lateral collision.

2. Complex scenario warning strategy.

In complex scenarios, two warnings may occur perceptually at the same time, requiring the driver to operate in both horizontal and vertical directions. According to the analysis of lane departure and horizontal warning scenarios in the single warning scenario, the warning flow chart of the two sub-warnings is shown in Fig. S6.

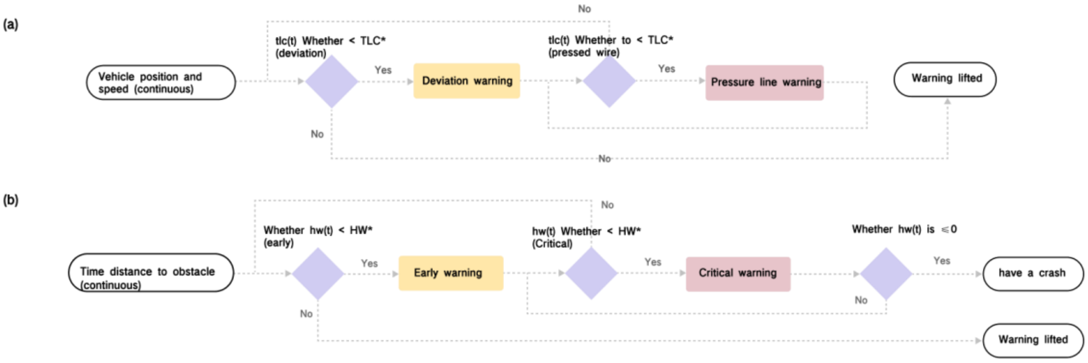

Figure S6. The flow chart of lane departure and lateral collision warning. (a) Lane departure warning flow chart. (b) Lateral collision warning flow chart.

In the lane departure warning system, the warning time is triggered by the threshold $TLC*$, and the early warning of deviation is gradually changed to the warning of line crossing. In the lateral collision warning system, the warning display time takes $HW*$ as the threshold, and the early collision warning is gradually changed to critical collision warning as the collision distance decreases.

According to the design framework of the AR-HUD warning interface proposed in this paper, since the horizontal and vertical warning can be discussed separately, there is no mutual interference and priority crowding between the two warning interfaces. The two warning interfaces can be presented at the same time and show the horizontal and vertical danger degree and acceleration deviation for the driver.

3. Prototype design of AR-HUD warning interface in complex scenario.

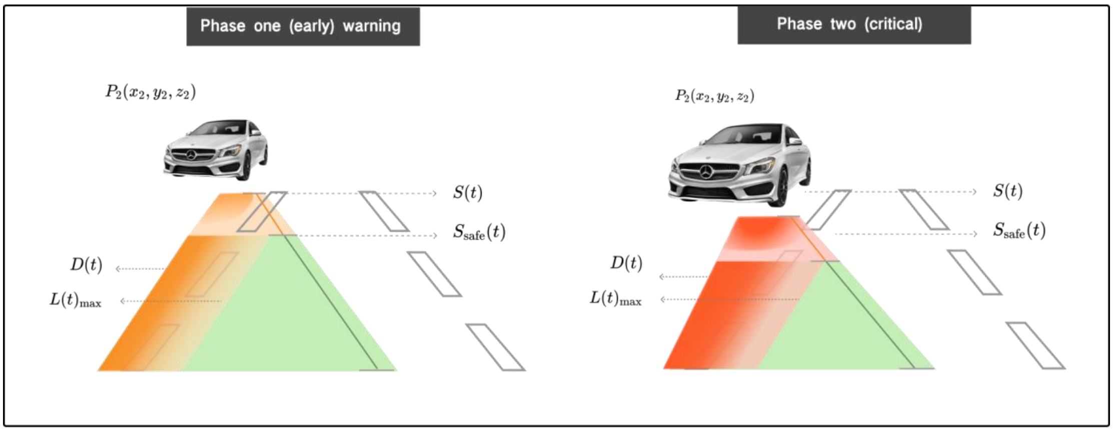

Figure S7. AR-HUD interface design for complex scenarios of lane departure and lateral warning.

Combined with the AR-HUD warning interface design framework and the analysis of lane departure and lateral warning in the single warning scenario, an integrated warning interface can be constructed for this complex scenario, as shown in Fig. S7. On the Y axis, the safety distance is expressed as $S_{safe}(t)$; on the X axis, the boundary value of the deviated side of the lane is the maximum allowable lateral deviation position $L(t)_{max}$. The interface designed by integration can focus the driver's attention on the dangerous areas, and by integrating the visual elements of KBB, RBB, and SBB information types, the driver's cognitive load can be balanced, which is conducive to the driver's faster perception and understanding of the current dangerous scenario. Meanwhile, the driver can quickly carry out the correct driving operation response.

Reference:

[S1] Gibson, J. J., & Crooks, L. E. (1938). A theoretical field-analysis of automobile-driving The American journal of psychology, 51(3), 453-471.

[S2] Rasmussen, J. (1983). Skills, rules, and knowledge; signals, signs, and symbols, and other distinctions in human performance models. IEEE transactions on systems, man, and cybernetics, (3), 257-266.
